# Molecular Anatomy of Synaptic and Extrasynaptic Neurotransmission Between Nociceptive Primary Afferents and Spinal Dorsal Horn Neurons

**DOI:** 10.3390/ijms26052356

**Published:** 2025-03-06

**Authors:** Miklós Antal

**Affiliations:** Department of Anatomy, Histology and Embryology, Faculty of Medicine, University of Debrecen, H-4032 Debrecen, Hungary; antal@anat.med.unideb.hu

**Keywords:** nociceptive information processing, spinal cord, nociceptive primary afferents, synaptic transmission, volume transmission, glutamate, neuropeptides, neurotrophins, endogenous opioids, presynaptic and postsynaptic receptors, monoamines

## Abstract

Sensory signals generated by peripheral nociceptors are transmitted by peptidergic and nonpeptidergic nociceptive primary afferents to the superficial spinal dorsal horn, where their central axon terminals establish synaptic contacts with secondary sensory spinal neurons. In the case of suprathreshold activation, the axon terminals release glutamate into the synaptic cleft and stimulate postsynaptic spinal neurons by activating glutamate receptors located on the postsynaptic membrane. When overexcitation is evoked by peripheral inflammation, neuropathy or pruritogens, peptidergic nociceptive axon terminals may corelease various neuropeptides, neurotrophins and endomorphin, together with glutamate. However, in contrast to glutamate, neuropeptides, neurotrophins and endomorphin are released extrasynaptically. They diffuse from the site of release and modulate the function of spinal neurons via volume transmission, activating specific extrasynaptic receptors. Thus, the released neuropeptides, neurotrophins and endomorphin may evoke excitation, disinhibition or inhibition in various spinal neuronal populations, and together with glutamate, induce overall overexcitation, called central sensitization. In addition, the synaptic and extrasynaptic release of neurotransmitters is subjected to strong retrograde control mediated by various retrogradely acting transmitters, messengers, and their presynaptic receptors. Moreover, the composition of this complex chemical apparatus is heavily dependent on the actual patterns of nociceptive primary afferent activation in the periphery. This review provides an overview of the complexity of this signaling apparatus, how nociceptive primary afferents can activate secondary sensory spinal neurons via synaptic and volume transmission in the superficial spinal dorsal horn, and how these events can be controlled by presynaptic mechanisms.

## 1. Introduction

After their activation by various acute and chronic noxious stimuli, nociceptive primary afferents conduct sensory signals from the peripheral tissue to the superficial spinal dorsal horn (laminae I–II). The central axon terminals of nociceptive primary afferents make glutamatergic synaptic contacts with spinal interneurons and projection neurons [1,2]. In addition to glutamate, in case of peripheral inflammation, neuropathy or the release of pruritogen agents, the central axon terminals of nociceptive primary afferents may also release a cocktail of other substances (neuropeptides, neurotrophins, endomorphin etc.) into the extracellular space [3,4]. Then, synaptically released glutamate excites postsynaptic neurons via the activation of postsynaptic ionotropic glutamate receptors [2]. Additionally, the nonsynaptically released neuropeptides, neurotrophins, and endomorphin, diffusing from the site of release, may exert various effects (excitation, disinhibition and inhibition) on a set of neurons in a tissue volume around the activated axon terminals, depending on the types of receptors they express and their sensitivity to the released substances [4,5]. Thus, the transmission of nerve signals from nociceptive axon terminals to spinal sensory neurons is a complex and collaborative performance of glutamatergic synaptic transmission and nonglutamatergic extrasynaptic volume transmission, generating an activation pattern of neurons in the superficial spinal dorsal horn that primarily defines how incoming nociceptive signals are further processed by spinal neurons, how projection neurons conducting various nociceptive signals to higher brain centers can be activated, and finally how the original nociceptive signals can be converted to sensations such as pain and itchiness by higher brain centers.

In addition, extrasynaptically released neurotransmitters may also activate astrocytes and microglia, and activated glial cells may also release additional biologically active substances that may act on presynaptic axons and postsynaptic neurons, increasing the complexity of the overall activation patterns of spinal sensory neurons [6,7]. Thus, neuron–glia–neuron bidirectional signaling mechanisms may substantially contribute to neural transmission between nociceptive primary sensory fibers and secondary spinal sensory neurons.

The functional properties of spinal nociceptive axon terminals and the release of neurotransmitters from them are under powerful presynaptic control. The spinal axon terminals of nociceptive primary afferents may express a wide range of presynaptic ionotropic and metabotropic receptors that can be activated in various ways depending on the actual composition of neurotransmitter substances within the synaptic cleft, and primarily in the extracellular space, released by nociceptive primary afferents themselves, spinal interneurons, and axons descending from the hypothalamus and brainstem [1,8,9,10,11,12,13]. The activation of presynaptic receptors may increase or attenuate neurotransmitter release from nociceptive axon terminals, thus attenuating or increasing the overall excitation level of neurons in the superficial spinal dorsal horn. Thus, without a thorough understanding of the presynaptic modulation of neurotransmitter release from nociceptive axon terminals, nociceptive information processing in the superficial spinal dorsal horn cannot be fully understood.

Because of its great importance in pain and itch perception, this review aims to provide an overview of the molecular anatomy of synaptic and extrasynaptic neurotransmission between nociceptive primary afferents and spinal dorsal horn neurons, including the molecular apparatus that is responsible for the presynaptic modulation of neurotransmitter release from nociceptive axon terminals. Pathophysiological mechanisms are sometimes mentioned but are not a primary focus, and potential therapeutic interventions are beyond the scope of this article.

## 2. The Primary Neurotransmitter of Nociceptive Primary Afferents Is Glutamate

**Figure 1:**
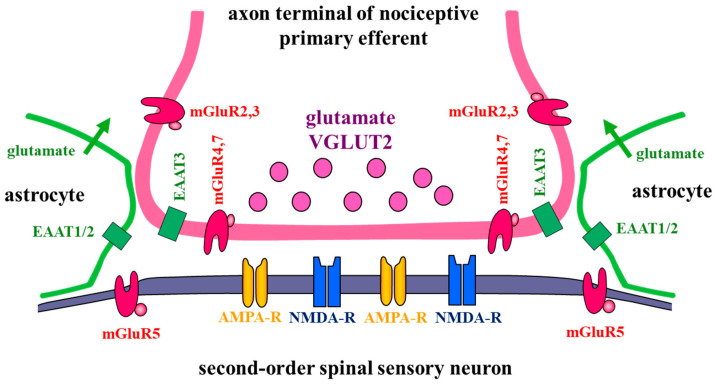
Schematic representation of the distribution of vesicular glutamate transporter 2 (VGLUT2), ionotropic (NMDA-R, AMPA-R) and metabotropic (mGluR2,3, mGluR4,7, mGluR5) glutamate receptors and excitatory amino acid transporters (EAAT1/2, EAAT3) at glutamatergic synapses formed by axon terminals of nociceptive primary afferents (magenta) and second-order spinal sensory neurons (blue) in laminae I–II of the superficial spinal dorsal horn. Astrocytic processes (green) closing the synaptic cleft from the side are also illustrated.

The fast excitatory neurotransmission from nociceptive primary afferents to second-order spinal sensory neurons is mediated by glutamate. The release of glutamate from the axon terminals of nociceptive primary afferents, the glutamatergic activation of postsynaptic neurons, and the retrograde glutamatergic presynaptic modulation of glutamatergic neurotransmission are dependent on a complex molecular apparatus, which has been discussed in detail in recent reviews [2,14,15]. For this reason, glutamatergic neurotransmission is reviewed only briefly here, and only three major components of this molecular system are discussed: vesicular glutamate transporters, glutamate receptors and nonvesicular excitatory neurotransmitter transporters (Figure 1).

### 2.1. Vesicular Glutamate Transporter 2

Although the vesicular glutamate transporter (VGLUT) content of the axon terminals of nociceptive primary afferents has long been unclear, there now seems to be a general agreement that glutamate is transported into small, clear synaptic vesicles of the spinal axon terminals of both C and Aδ nociceptive primary afferents by the VGLUT2 isoform of VGLUTs, which exist in three isoforms (VGLUT1-3) in the central nervous system [16,17]. The uncertainty concerning the VGLUT2 content of the spinal axon terminals of nociceptive primary afferents results from the findings demonstrating that glutamatergic axon terminals in laminae I–II of the spinal dorsal horn showed strong VGLUT2 immunoreactivity, and dorsal rhizotomy caused only a slight reduction in VGLUT2 immunostaining [18]. In addition, VGLUT2 immunoreactivity was detected only in a small proportion of central axon terminals of nociceptive primary afferents identified by transganglionic labeling or chemical markers [19,20]. However, other studies have shown that virtually all neurons in the rat dorsal root ganglia (DRG) that expressed CGRP or TRPV1 or showed IB4-binding, which are markers of different populations of nociceptive DRG neurons, were simultaneously positively stained for VGLUT2 [21,22]. Moreover, the analysis of VGLUT2 conditional knockout mice clearly revealed that VGLUT2-dependent glutamate release from nociceptive primary afferents plays a crucial role in neuropathic pain [22]. Thus, the only plausible explanation for the misleading data obtained from the immunohistochemical staining experiments is that glutamatergic axon terminals of spinal origin may contain VGLUT2 in much greater quantities than the axon terminals of nociceptive primary afferents in laminae I–II of the spinal dorsal horn. In addition, the level of VGLUT2 in some as-yet unidentified populations of axon terminals of nociceptive primary afferents is below the sensitivity threshold of presently available immunohistochemical methods.

### 2.2. Glutamate Receptors

Synaptically released glutamate acts on ionotropic and metabotropic receptors, many of which are robustly expressed in the spinal dorsal horn, including synapses made by nociceptive primary afferents on spinal neurons [1,2,23].

#### 2.2.1. Ionotropic Glutamate Receptors

Ionotropic glutamate receptors are classified into three groups: *N*-methyl-d-aspartate (NMDA), α-amino-3-hydroxy-5-methyl-4-isoxazole propionic acid (AMPA) and kainate receptors. All of them are tetrameric ion channels formed by various protein subunits in different combinations. Multiple subunits have been cloned in each of these receptor types, as follows: the NR1, NR2A-D, and NR3A-B subunits for NMDA receptors; the GluR1-4 subunits for AMPA receptors; and the GLUK1-5 subunits for kainite receptors [14,15]. NMDA receptors are obligatory heteromers. These receptors must contain at least one NR1 subunit and two or three NR2 and NR3 subunits. AMPA and kainate receptors can assemble as either homomers or heteromers. Independent of the subunit composition, the NMDA receptor channel shows predominant permeability to Ca^2+^, and is blocked in a voltage-dependent manner by a Mg^2+^ ion, which is removed from the channel when the neuron is sufficiently depolarized by the opening of other depolarizing cation ion channels, including AMPA receptors. AMPA receptor channels show high permeability to Na^+^ ions. In addition, AMPA receptors that do not contain GluR2 subunits are permeable to Ca^2+^ [24,25]. The kainate receptor channel is permeable to both Na^+^ and K^+^. In the case of specific subunit compositions, it may also show a slight permeability to Ca^2+^ [26,27]. NMDA and AMPA receptors in the spinal dorsal horn are involved in various physiological events, including synaptic plasticity [2,28,29]. Kainate receptors, on the other hand, make only minimal, if any, contributions to synaptic transmission from nociceptive primary afferents to spinal neurons [30]. For this reason, this review focuses exclusively on NMDA and AMPA receptors.

Among the three NMDA receptor subunit families, the NR1, NR2A and NR2B proteins are the most important in the construction of functional NMDA receptors [2,15,31]. Concerning the subunit composition of AMPA receptors, the GluR1 and GluR2 subunits are heavily expressed, whereas the other two subunits have been detected in minimal quantities in the superficial spinal dorsal horn (laminae I–II) [32,33]. Thus, studies regarding the distribution of NMDA and AMPA receptors in postsynaptic membranes have focused on the NR1, NR2, GluR1 and GluR2 subunits [33,34,35]. These studies have revealed that the postsynaptic membranes of virtually all (96%) glutamatergic synapses in laminae I–II, including those that are made by nociceptive axon terminals, contain NMDA receptors, and are most likely constructed from NR1 and NR2B subunits [33,35]. All postsynaptic membranes positive for NMDA receptors also express AMPA receptors in laminae I–II of the rat spinal gray matter [35]. In addition, the distribution of both AMPA and NMDA receptors within postsynaptic membranes is rather homogeneous, suggesting the possibility of direct and very effective collaboration between AMPA and NMDA receptor mechanisms [35].

Concerning the expression of the individual subunits of AMPA receptors in the superficial spinal dorsal horn, it has been shown that virtually all postsynaptic membranes (99%) possess the GluR2 subunit, and approximately 87% of them also possess the GluR1 subunit [35]. In postsynaptic membranes in which both GluR1 and GluR2 were detected, the numbers of the individual subunits varied over a wide range, and were independent of each other. There were synapses with high GluR2 expression and low GluR1, and others with low GluR1 and high GluR2 expression [35]. This finding indicates that the GluR1 and GluR2 subunits may form both homomeric and heteromeric; thus, both Ca^2+^-permeable (lacking the GluR2 subunit) and Ca^2+^-impermeable (possessing the GluR2 subunit) AMPA receptors [36,37] and the ratio between Ca^2+^-permeable and Ca^2+^-impermeable AMPA receptors may vary widely, even within individual postsynaptic membranes [35].

#### 2.2.2. Metabotropic Glutamate Receptors

Metabotropic glutamate receptors (mGluRs) modulate rather than mediate synaptic transmission. There are eight subtypes of mGluRs (mGluR1-8), all of which belong to the C class of G-protein-coupled receptors [1,23]. They are divided into three groups according to their molecular structure, pharmacological properties and coupled intracellular signaling pathways [38,39]. Some members of all three groups of mGluRs are localized at synapses made by nociceptive primary afferents on secondary spinal neurons in laminae I–II, but their synaptic localization, and thus their effects on glutamatergic transmission, greatly differ.

Group I mGluRs (mGluR1 and mGluR5) are coupled to G_q/11_ proteins, and activate phospholipase C (PLC), resulting in the hydrolysis of phosphatidylinositol 4,5 biphosphate (PIP2) into inositol 1,4,5 triphosphate (IP3) and diacylglycerol (DAG). Both members of group I mGluRs (mGluR5 and mGluR1) have been identified as postsynaptic receptors [1,40]. mGluR5 is strongly expressed in laminae I–II, especially in lamina IIi [41,42,43], whereas mGluR1 expression is very low in laminae I–II [42]. Thus, mGluR5 seems to be the main postsynaptic mGluR receptor in laminae I–II. However, unlike NMDA and AMPA receptors, mGuR5 is localized in the periphery of the postsynaptic membranes of synapses made by nociceptive primary afferents, establishing a so-called perisynaptic location [41,42]. This suggests that mGluR5 can be activated only by the extensive release of glutamate from presynaptic axons. The biological importance of this special localization of mGluR5 in nociceptive synaptic transmission will be discussed later.

Group II mGluRs (mGluR 2 and mGluR3) and group III mGluRs (mGluR4, mGluR6, mGluR7 and mGluR8) are coupled to G_i/o_ proteins. The activation of these receptors inactivates adenylyl cyclase (AC), and thus decreases the formation of cyclic adenosine monophosphate (cAMP). In addition, they inhibit voltage-sensitive Ca^2+^ channels and increase the probability of inward-rectifier K^+^ channel opening [1,23,44,45,46]. Group II and Group III mGluRs are presynaptic receptors, the activation of which inhibits transmitter release from presynaptic axon terminals. Although both the mGluR II and mGluR III receptors depress synaptic activity, their biological functions can differ substantially, because they show major differences in their distributions within the cell membrane of presynaptic axon terminals [1,23]. Group III mGluRs are localized on presynaptic membranes, whereas Group II mGluRs are extrasynaptic, keeping a considerable distance from the synaptic appositions [1,23]. The expressions of mGluR 2/3, mGluR4 and mGluR7 on the axon terminals of nociceptive primary afferents in laminae I–II have been identified [1,41,47,48,49]. Owing to their distant localization from the synaptic cleft, mGluR 2/3 receptors cannot be activated by glutamate released by the axon terminals of nociceptive primary afferents; thus, they may bind the glutamate released by adjacent astrocytes [50] and can influence synaptic transmission only indirectly. However, mGluR4 and mGluR7, located in the presynaptic membrane, can be major constituents of the molecular machinery of synapses made by the central axon terminals of nociceptive primary afferents. They can serve as “rate-limiting” autoreceptors, regulating the quantity of glutamate released from the axon terminals [1,23].

Importantly, however, mGluR7 has been shown to be expressed by only a subset of nociceptive primary afferents [47], and this may be true for the other mGluRs as well [51]. Thus, the actual compositions of pre- and postsynaptic mGluR receptors at individual synapses need further verification. It is conceivable that ubiquitous ionotropic glutamatergic transmission can be modulated by mGluR receptors differently at synapses made by functionally different subsets of nociceptive primary afferents.

### 2.3. Excitatory Amino Acid Transporters

Excitatory amino acid transporters (EAATs) are localized on the cell membranes of both neurons and glial cells. They bind to glutamate with high affinity and transport it from the extracellular space to the cytoplasm, along with the transport of three Na^+^ ions and one H^+^ ion, and the countertransport of one K^+^ ion [52]. Five isoforms of EAATs (EAAT1-5) have been identified in the central nervous system [23,52,53], and three of the isoforms (EAAT1-3) have been discovered in the spinal dorsal horn [53,54,55,56]. EAAT1 (or GLAST) and EAAT2 (or GLT1) are expressed on astrocytes, predominantly in their small processes [54,55,56], which attach to synapses, virtually closing the synaptic cleft from the side. By establishing this perisynaptic location, EAAT1 and EAAT2 limit, if not completely block, glutamate spillover from the synaptic cleft to the surrounding extracellular space. The glial EAATs uptake 95% of the excess glutamate in the synaptic cleft, according to certain calculations [53]. Although to a limited extent, neuronal EAAT3 (or EAAC1) may also contribute to the uptake of glutamate in the synaptic cleft. EAAT3 has been detected not only in the neuropil in laminae I–II, but also in small neurons within the dorsal root ganglia (DRG) [53,54,55,57]. Some of the EAAC1-positive DRG neurons also showed positive immunostaining for calcitonin gene-related peptide (CGRP), or were labeled with isolectin B4 (IB4) [54,57], which are markers of peptidergic and nonpeptidergic nociceptive primary afferents. In addition, some puncta in the superficial spinal dorsal horn that were positively immunostained for EAAT3 were also positive for CGRP or labeled with IB4, and dorsal rhizotomy resulted in a decrease in EAAT3 immunoreactivity in laminae I–II [54]. Thus, it is likely that EAAT3 molecules are localized on the presynaptic membrane of synapses between nociceptive primary afferents and spinal neurons, and that they may modulate the time course of glutamatergic nociceptive synaptic transmission.

## 3. Glutamatergic Neurotransmission Can Be Modulated by the Simultaneous Release of Neuropeptides and Neurotrophins

### 3.1. Neuropeptides

**Figure 2:**
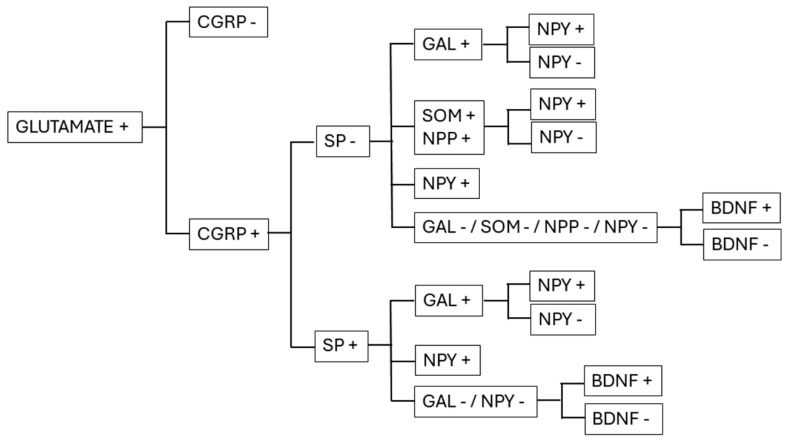
Illustration of possible colocalizations of glutamate, neuropeptides and brain-derived neurotrophic factor in the axon terminals of nociceptive primary afferents in laminae I–II of the superficial spinal dorsal horn. The illustration is based on a wide range of published experimental findings cited in the text of the article. CGRP—calcitonin gene-related peptide; SP—substance P; GAL—galanin; SOM—somatostatin; NPP—natriuretic polypeptide; NPY—neuropeptide Y; BDNF—brain-derived neurotrophic factor.

Myelinated Aδ and nonmyelinated C nociceptive fibers terminating in laminae I and IIo can simultaneously release glutamate and neuropeptides. For this reason, they are referred to as peptidergic nociceptive primary afferents. The other population of nociceptive nonmyelinated C fibers terminating in lamina IIi can release only glutamate without the corelease of neuropeptides, and is referred to as nonpeptidergic nociceptive primary afferents [3] (Figure 2).

Neuropeptides released by primary afferents are synthetized in the cell bodies of primary sensory neurons within the DRG as precursor proteins. The precursors then undergo posttranslational modifications, and the final forms of the peptides are enclosed in large granular (dense core) secretory vesicles (LGVs). LGVs are transported along the axons of DRG neurons and ultimately accumulate in both the peripheral and central axon terminals of DRG neurons [4]. In the synaptic terminals of central axons, LGVs are present in much smaller numbers than the glutamate-storing small clear vesicles [58], and they are also located farther from the presynaptic membrane than most small clear vesicles. For this reason, much higher intraaxonal Ca^2+^ concentrations and firing frequencies are required for LGVs to release neuropeptides compared to glutamate [5]. In addition, only a limited quantity, if any, of the neuropeptides can be released into the synaptic cleft. Typically, they are discharged into the extracellular space. Because of the lack of high-affinity uptake mechanisms and because the affinity of neuropeptide receptors for their ligands is very high (neuropeptides can activate their receptors at very low nanomolar concentrations), following local release, neuropeptides can diffuse widely within the extracellular space, and can bind to specific extrasynaptic receptors located several tens of a micrometer from the site of release [4,5,58]. Thus, peptidergic neurotransmission can be best described as volume transmission.

Neuropeptide receptors are G protein-coupled metabotropic receptors whose activation initiates complex signaling cascades resulting in protein phosphorylation and the regulation of gene expression. In this way, neuropeptides modulate the biophysical parameters of the postsynaptic neurons and the sensitization of specific ion channels in a slow-to-develop and long-lasting (from seconds to minutes) manner. Thus, although neuropeptides do not act together with glutamate at the postsynaptic membranes of synapses, they can modulate glutamate-evoked postsynaptic responses in indirect but very effective ways, which enable slow (100–500 ms) postsynaptic currents in addition to the fast (2–5 ms) postsynaptic depolarizations evoked by glutamate alone [4,5,58]. In addition, although this is beyond the scope of the present review, it is also important to note that unlike glutamate, neuropeptides may activate multiple neurons in the spinal dorsal horn upon their release from a single presynaptic bouton [59].

#### 3.1.1. Calcitonin Gene-Related Peptide (CGRP)

CGRP is a 37-amino-acid peptide that is present in two isoforms, CGRPα and CGRPβ [60]. In laminae I–IIo of the spinal dorsal horn, both isoforms have been detected, and two-thirds and one-third of the total CGRP content are produced by CGRPα and CGRPβ, respectively [61]. Although they are encoded by different genes, the two isoforms differ from each other in only three amino acids. Owing to this high similarity in molecular architecture, the physiological and pharmacological events evoked by the two isoforms cannot be distinguished from each other, and anti-CGRP antibodies recognize both isoforms [4]. Concerning the expression of CGRP in the axon terminals of nociceptive primary afferents in the superficial spinal dorsal horn, there is general agreement that it is expressed in virtually all peptidergic nociceptive primary afferents in laminae I–IIo, whereas the nonpeptidergic terminals in lamina IIi do not express the peptide [3].

The canonical CGRP receptor is a metabotropic calcitonin receptor-like receptor (CLR) coupled to a G_s_ protein. However, the CLR-G_s_ complex is nonfunctional and must be associated with an isoform of a single transmembrane domain protein called receptor activity-modifying protein (RAMP1). After binding CGRP, the CLR–RAMP1-G_s_ complex activates AC, which leads to the consecutive production of cAMP and the activation of protein kinase A (PKA). The activation of PKA and other kinases results in the phosphorylation of CREB, K^+^ and Ca^2+^ ion channels, as well as AMPA receptors, promoting neuronal excitability and synaptic plasticity [4,5,62].

Early autoradiographic receptor binding studies have revealed that CGRP receptors are densely arranged in the laminae I–II of the spinal dorsal horn [63,64]. These findings have been reinforced by later studies detecting mRNAs encoding the proteins of the receptor complex and several immunocytochemical experiments that were performed to directly detect the CLR and RAMP1 proteins in both the DRG and the spinal dorsal horn [65,66,67]. The distribution of receptor protein immunoreactivity closely matched with CGRP immunoreactivity in DRG neurons [66,67]. Colocalization between receptor proteins and the GluR2 subunit of AMPA receptors has also been revealed in neurons within the spinal dorsal horn [65,68]. The subcellular localization of CGRP receptors in laminae I–II, however, still requires accurate high-resolution mapping. The difficulty in determining the precise subcellular localization of CGRP receptors is that they require the simultaneous and reliable detection of the CLR and RAMP1 proteins with well-characterized antibodies. However, most antibodies that have been used until now have not been appropriately validated. Thus, the experimental data available now do not necessarily reflect the precise subcellular distribution of CGRP receptors. Keeping this uncertainty in mind, according to the available experimental data, CGRP receptors are likely distributed in both post- and presynaptic locations within the synapses between peptidergic nociceptive afferents and spinal neurons, and they enhance glutamatergic synaptic transmission at both locations [4,5,10,62].

CGRP signaling in laminae I–II, however, can be much more complex than the described interaction between CGRP and the CLR–RAMP1 receptor complex. There are three isoforms of RAMPs (RAMP1, RAMP2 and RAMP3), and CLR can form functional receptor complexes with all three isoforms. Although the CLR–RAMP1 complex has the highest affinity for CGRP [4,69], the CLR–RAMP2 (AM1 receptor) and CLR–RAMP3 (AM2 receptor) complexes can also be effectively activated by the CGRP-like peptide adrenomedullin (AM), the expression of which has been reported both in DRG and spinal neurons [70,71], indicating that AM may act in parallel with CGRP in pain processing [70,71].

CGRP and CGRP-like peptide signaling in laminae I–II is even more complex, as in addition to CLR, a CGRP-sensitive receptor has been identified. This second receptor has been named the calcitonin receptor (CTR), which also forms complexes with all three RAMPs. Because these complexes can also bind amylin, a peptide closely related to CGRP, they are referred to as amylin receptors (AMY1, AMY2 and AMY3) [60,72,73]. The CTR–RAMP1 complex (AMY1 receptor) can be considered as a second CGRP receptor, because it binds CGRP with high affinity [73,74,75]. Although AMY2 and AMY3 receptors are less sensitive to CGRP, they can still bind amylin, which is expressed in a subpopulation of CGRP-containing DRG neurons and their spinal axon terminals, with high affinity [4,73]. Surprisingly, the pharmacological modulation of AMY receptors at the level of the spinal cord induced both pro- and antinociceptive effects [76,77,78].

#### 3.1.2. Substance P (SP)

SP is an 11-amino-acid-long member of the tachykinin family of neuropeptides [5,58,79]. It is expressed by 40–50% of CGRP-containing peptidergic nociceptive primary afferents [80]. Thus, peptidergic nociceptive primary afferents can be subdivided into two subgroups, CGRP+/SP+ and CGRP+/SP− (Figure 2). SP is costored with CGRP within the same LGVs [58], indicating that SP and CGRP are released together from the SP-positive peptidergic nociceptive axon terminals, and that the two peptides act simultaneously on their target neurons in the superficial spinal dorsal horn [81]. The collaboration between the two neuropeptides is even more remarkable because there is evidence that, in addition to their actions discussed above, CGRP receptors stimulate an intercellular signaling pathway resulting in an increased level of SP (NK) receptor expression in postsynaptic neurons [82]. SP-expressing primary afferents may also contain and release neurokinin A, but this cannot substantially alter the effect of SP because it is 250 times less potent than SP is [5].

SP acts on metabotropic neurokinin receptors (NKRs), which are present in three isoforms in the central nervous system, NK1R, NK2R, and NK3R. Two of the three isoforms, NK1R and NK3R, are distributed almost exclusively in lamina I and lamina II of the spinal dorsal horn, respectively [83,84,85]. Both NK1Rs and NK3Rs are expressed by the somato-dendritic membrane compartments of specific sets of spinal neurons, but they are not expressed in DRG neurons or axon terminals in laminae I–II. In lamina I, NK1Rs are expressed by the cell bodies and dendrites of supraspinally projecting neurons [83]. Most NK3R-expressing neurons in lamina II also express nitric oxide synthase (NOS) or µ-opioid receptor (MOR1) according to immunostaining results [84].

All types of NKRs are coupled to Gq-proteins, through which they activate phospholipase C (PLC), resulting in the production of IP3 and DAG, and a consecutive elevation of intracellular Ca^2+^ levels. These events activate various kinases, phosphorylating and sensitizing AMPA and NMDA ion channels, thus enhancing the postsynaptic effects of glutamatergic synaptic transmission [85,86].

The increased level of Ca^2+^ in postsynaptic neurons may also activate cyclo-oxygenase, resulting in the synthesis of prostaglandin E2 (PGE2). PGE2 can diffuse out of postsynaptic neurons and may activate presynaptic prostaglandin receptors (EPs) [87]. PGE2 has four receptors, EP_1–4_, which are metabotropic receptors coupled to different G proteins [87,88]. EP_1_ receptors are coupled to G_q/11_, and EP_2_ and EP_4_ receptors are coupled to G_s_ protein. EP_3_ receptors have three splice variants, EP_3α_, EP_3β_ and EP_3γ_. EP_3α_ and EP_3β_ receptors are coupled to G_i_ protein, but EP_3γ_ receptors can be coupled to both G_s_ and G_i/o_ proteins [89,90]. Early autoradiographic studies revealed that the EP2 receptor is expressed by spinal interneurons, whereas the EP_1_, EP_3_ and EP_4_ receptors are expressed in both small and large DRG neurons [91]. Unfortunately, there are still no direct experimental data on whether one or more of these receptor proteins can be transported to the central axon terminals of nociceptive primary afferents. An early study performed on synaptosomes isolated from the rat spinal dorsal horn revealed that the application of PGE2 substantially increased neurotransmitter release from synaptosomes in a Ca^2+^-dependent manner [92]. In a more recent study, although EP_1_ receptor mRNA could not be detected in spinal cord tissues, the application of an EP_1_ receptor antagonist markedly attenuated PGE2-induced hyperalgesia and suppressed the PGE2-induced increase in intracellular Ca^2+^ in the dorsal horn [93]. These findings indicate that the release of SP from the axon terminals of peptidergic nociceptive primary afferents may activate a positive feedback loop, increasing further neurotransmitter release from nociceptive axon terminals through PGE2-EP receptor signaling pathways.

#### 3.1.3. Somatostatin (SOM) and Natriuretic Polypeptide (NPP)

Although there are two biologically active isoforms of SOM (SOM-14 and SOM-28), only the SOM-14 isoform has been isolated from the central nervous system, including laminae I–II of the spinal dorsal horn. SOM-14 is expressed in a unique group of peptidergic nociceptive primary afferents that do not overlap with the SP-containing population [94] (Figure 2). In addition, transcriptomic and immunohistochemical studies have demonstrated that SOM is coexpressed with another neuropeptide, NPP, in both DRG neurons and their spinal axon terminals in laminae I–II [95,96,97]. NPP is present in two isoforms in the spinal dorsal horn, as follows: (1) NPPA, which is a 28-amino-acid-long peptide that is expressed at high levels in mice, rats, and humans, and (2) NPPB, a 32-amino-acid-long peptide that is expressed only in mice at high concentrations [98,99].

Four (sst1–sst4) of the five cloned SOM receptors have been detected in the mammalian central nervous system [100]. SOM-14 can bind to all four subtypes of the receptor. Most attention has been given to the investigation of sst2 because it is the most abundant SOM receptor in both the brain and the spinal cord. There are two splice variants of sst2, sst2A and sst2B. Both have been detected in the rodent brain, but only sst2A has been detected in human tissues [101]. In the rodent spinal cord, sst2A is expressed only in the superficial spinal dorsal horn, whereas sst2B is faintly expressed in this region and is distributed throughout the gray matter. Early studies have provided strong evidence that sst2A in laminae I–II is expressed exclusively in the somatodendritic membrane of spinal neurons [102], and has never been detected in primary afferents.

All sst receptors are metabotropic receptors associated with Gi/o proteins [101,103]. The activation of these receptors leads to the inactivation of AC, the suppression of cAMP, a decrease in the cytoplasmic Ca^2+^ concentration caused by the inhibition of Ca^2+^ channels, and the opening of inward-rectifier K^+^ channels. As a result, the activation of sst receptors results in the hyperpolarization of postsynaptic neurons [97,101].

Natriuretic polypeptide receptors are present in three isoforms, NPRA, NPRB and NPRC, but NPRA (also called NPR1) is the principal receptor of both NPPA and NPPB [98,99,104]. NPRA is a dimeric metabotropic receptor with an intracellular guanylyl cyclase catalytic domain that catalyzes the synthesis of cGMP [105,106] and activates postsynaptic neurons [107].

The complete coexpression of SOM and NPP suggests that they are released together and simultaneously from the same set of primary afferents, but the expressions of the sst2A and the NPR1 receptors are completely segregated from each other on spinal interneurons in laminae I–II. The sst2A receptor is expressed exclusively on inhibitory dynorphin-containing GABAergic interneurons [97,108,109]. Because the activation of the sst2A receptor results in hyperpolarization, thus inhibiting dynorphin/GABA release from postsynaptic neurons, the release of SOM from primary afferents ultimately results in the disinhibition of neurons that are innervated by the dynorphin/GABA neurons [97,109]. On the other hand, NPR1 is expressed in a limited subset of excitatory neurons, primarily in lamina I, all of which contain gastrin-releasing peptide (GRP) [104,107]. Interestingly, it is likely that the neurons disinhibited by the activation of sst2A receptors are the same GRP-containing cells that are excited by the activation of NPR1 receptors [97].

#### 3.1.4. Galanin (GAL)

GAL is a 29-amino-acid-long neuropeptide synthesized by small- and medium-sized DRG neurons [110]. In laminae I–II, GAL has been detected in a proportion of axon terminals that contain CGRP. Some of these axon terminals also showed positive immunostaining for SP, in that GAL was stored together with CGRP and SP in the same large dense-core synaptic vesicles [111], indicating that GAL can be released with CGRP and, in some cases, with SP from nociceptive primary afferents (Figure 2).

Three types of G protein-coupled metabotropic galanin receptors have been identified, namely, GalR1, GalR2 and GalR3. Cellular localization studies have established that in laminae I–II of the spinal dorsal horn, GalR1 receptors are expressed on postsynaptic glutamatergic spinal neurons [112], whereas GalR2 receptors are detected almost exclusively in CGRP-containing presynaptic primary afferents. In addition to GalR2 receptors, nociceptive primary afferents may also exhibit a weak GalR1 receptor expression [110,113,114,115]. The expression of the GalR3 receptor in the spinal dorsal horn is very low, if detectable [116]. GalR1 receptors are coupled with Gi/o proteins, the activation of which inactivates AC and inhibits cAMP production. Associated with the activation of GalR1 receptors, GAL also opens inward-rectifier K^+^ channels, leading to the hyperpolarization of postsynaptic neurons [110,117]. GalR2 receptors are unique, because they can be associated with both Gi/o and Gq/11 proteins. Thus, although the activation of GalR2 receptors also inactivates AC and inhibits the production of cAMP, it activates phospholipase C (PLC), increasing the production of IP3, DAG and the intracellular concentration of Ca^2+^. The increased level of Ca^2+^ then enhances glutamate release from presynaptic nerve terminals [118]. Because of the antagonistic effects of GalR1 and GalR2, the release of galanin from nociceptive axon terminals may exert various effects on local spinal neuronal circuits [118]. Moreover, the proper evaluation of the effects of GAL on spinal nociceptive neural circuits is even more complex, as GAL is presumably released simultaneously with glutamate, CGRP, and sometimes also with SP.

#### 3.1.5. Neuropeptide Y (NPY)

NPY is a 36-amino-acid-long peptide synthesized by a large population of DRG neurons including small- and medium-sized cell bodies. In the superficial spinal dorsal horn, NPY immunoreactive axon terminals also express CGRP [111]. In addition to CGRP, some NPY-containing primary afferents also express GAL [111], and electrophysiological and pharmacological studies indicate that some may also express SP, SOM, and NPP [119,120]. Thus, because of the lack of proper neurochemical delineation, the neuropeptide expression profile of NPY-containing nociceptive primary afferents can be extremely variable. Some of the possible expression profiles include (1) glutamate+, CGRP+, NPY+, (2) glutamate+, CGRP+, SP+, NPY+, (3) glutamate+, CGRP+, SP+, GAL+, NPY+, (4) glutamate+, CGRP+, GAL+, NPY+, (5) glutamate+, CGRP+, SOM/NPP+, and NPY+ (Figure 2).

NPY binds to at least five different receptors (Y1-Y5 receptors), but only the Y1 and Y2 receptors have been detected in the superficial spinal dorsal horn [121]. The Y1 and Y2 receptors show almost complete segregation in laminae I–II. The finding that dorsal rhizotomy completely eliminated Y2 receptor immunoreactivity from the spinal dorsal horn [122] led to the conclusion that the Y2 receptor in laminae I–II is confined to the central terminals of primary afferents, and is not expressed by spinal neurons [95,122], although a recent analysis has shown low Y2 receptor mRNA expression in some dorsal horn interneurons [108]. Y2 receptors are expressed in both peptidergic and nonpeptidergic nociceptive primary afferents [121,122]. On the other hand, it is generally agreed that the Y1 receptor is highly expressed in spinal interneurons [11,121,123], which also express VGLUT2, but not PAX2 [123,124], indicating that almost all Y1 receptor-expressing interneurons in the dorsal horn are excitatory. Transgenic and morphological approaches have revealed that Y1 receptor-positive interneurons in lamina II resemble the morphology of central and radial cells [124,125]. In lamina I, Y1-expressing neurons are large, and some of them have been identified as supraspinally projecting NK1 receptor-expressing neurons [11,121,124]. In addition, Y1 receptors in laminae I–II have been detected on the dendrites of neurons, the cell bodies of which are located at the border between laminae III and IV. With respect to their neurochemical characteristics, a large population of Y1 receptor-positive neurons in lamina II also expresses SOM [11,122,123,126], and some of these neurons may also contain calbindin, calretinin and gastrin-releasing peptide [11,124].

All Y receptors are Gi/o protein-coupled metabotropic receptors. The activation of both Y1 and Y2 receptors leads to the inactivation of AC, attenuating cAMP production. In addition, NPY binding to Y1 and Y2 receptors inhibits Ca^2+^ channels and activates inward-rectifier K^+^ channels in the plasma membrane [11,120,121]. For this reason, the current consensus is that NPY hyperpolarizes excitatory spinal interneurons and attenuates neurotransmitter release from the axon terminals of nociceptive primary afferents, thus blocking action potential discharges both pre- and postsynaptically [11,120,124].

#### 3.1.6. Other Neuropeptides

Early immunohistochemical studies indicated that, in addition to the neuropeptides described above, nociceptive primary afferents may also express cholecystokinin, vasoactive intestinal polypeptide, dynorphin, and endorphin. However, more detailed investigations have shown that these peptides may be present in the cell bodies of DRG neurons and peripheral axons, but their expressions in the spinal axon terminals of nociceptive primary afferents are very low, if they are expressed at all. For this reason, their contribution to neurotransmission between nociceptive primary afferents and secondary sensory neurons in the spinal dorsal horn is not discussed in this review.

### 3.2. Neurotrophins

Neurotrophins are a family of secreted proteins that influence the generation, proliferation, differentiation, and survival of neurons, as well as the axonal and dendritic growth in both the peripheral and central nervous systems [127,128,129]. In addition to acting as growth factors, neurotrophins also modulate the expressions of various biosynthetic enzymes, transmitter substances, and ion channels, and thus contribute to synaptic plasticity [129,130,131]. In mammals, the neurotrophin family consists of four members: nerve growth factor (NGF), brain-derived neurotrophic factor (BDNF), neurotrophin-3 (NT-3) and neurotrophin-4 (NT-4; also known as NT-4/5) [129,132]. Neurotrophins are synthesized in three steps, as follows: (1) initially, pro-neurotrophins are produced (210–270-amino-acid-long proteins); (2) pro-neurotrophins are cleaved into the final amino acid sequence (approximately 120-amino-acid-long proteins); and (3) the neurotrophin molecules form homodimers, which are ultimately enclosed in dense-core vesicles [133,134,135,136].

Neurotrophins bind to two classes of receptors, tyrosine kinase receptors (Trk receptors; also known as tropomyosin-related kinase receptors) and p75 receptor. Trk receptors bind specific neurotrophins with high affinity; NGF binds to the TrkA receptor, BDNF and NT-4 bind to the TrkB receptor, and NT-3 binds to the TrkC receptor. The p75 receptor is a pan-neurotrophin receptor that binds all neurotrophins, including pro-neurotrophins, with low affinity [128,129]. After binding to specific neurotrophins, the intracellular tyrosine residues of Trk receptors undergo autophosphorylation. Phosphorylated tyrosine residues can bind and activate various signaling molecules, the activation of which initiates multiple intracellular signal transduction pathways promoting cell survival and differentiation during development and synaptic plasticity in adults [128,132,137,138]. The p75 receptor may associate with Trk receptors, enhancing the actions of the actual Trk receptor. If p75 receptors are expressed alone, they can activate specific intracellular signaling pathways, leading to apoptotic cell death [12,130,139].

Concerning the distribution of neurotrophins, NT3 has been found in muscle spindle afferents, but it has not been detected in the adult central nervous system [128]. NT-4 has been shown to be synthesized by neurons in the sympathetic ganglia, DRG, and spinal motoneurons [140]. However, it has never been found in neurons that participate in spinal nociceptive information processing, affirming the notion that NT4-TrkB signaling does not contribute to nociception [128]. There is a general agreement that NGF is a peripheral neurotrophin [128], and its TrkA receptor is strongly expressed in small- and medium-sized DRG neurons, which synthesize and transport BDNF to the superficial spinal dorsal horn [12,141,142]. BDNF immunoreactivity has been detected in most, if not all, TrkA-expressing DRG neurons [141]. Moreover, there is strong evidence that BDNF synthesis in DRG neurons is dependent on the activation of TrkA receptors [12,128]. These findings suggest that the peripheral production of NGF induced by inflammatory and neuropathic processes activates BDNF synthesis in small- and medium-sized DRG neurons via TrkA receptor-initiated signaling events. BDNF is then transported to the superficial spinal dorsal horn by the central axons of activated DRG neurons, where it is released from axon terminals and acts on spinal neurons expressing TrkB receptors [12,143]. Thus, one may conclude that, concerning neurotrophins, only the NGF-TrkA-BDNF-TrkB signaling apparatus can modulate synaptic transmission between nociceptive primary afferents and spinal neurons in laminae I–II of the spinal dorsal horn [144].

#### BDNF

BDNF-synthesizing TrkA receptor-positive DRG neurons also express CGRP, and many of them are also positive for SP [145]. The same colocalization was also detected in the central axon terminals of nociceptive primary afferents in which BDNF has been found in large dense-core synaptic vesicles accompanied by CGRP, and, in many cases, also by SP [146,147,148]. The colocalization of BDNF with other neuropeptides has never been reported (Figure 2). The expression level of BDNF under physiological conditions is low, but peripheral inflammatory and neuropathic processes associated with NGF production can induce substantial increases in BDNF expression both in DRG neurons and in axon terminals in laminae I–II of the spinal dorsal horn via the activation of TrkA receptors in DRG neurons [149].

In the superficial spinal dorsal horn, TrkB receptors are expressed in both post- and presynaptic locations. They have been found in the somatodendritic membranes of at least half of the neurons, including spinothalamic projection neurons, and in approximately one third of the axon terminals of nociceptive primary afferents in laminae I–II of the spinal dorsal horn [128,143,148]. Among TrkB-positive primary afferents, both peptidergic and nonpeptidergic axon terminals have been identified [148]. The binding of BDNF to TrkB receptors simultaneously activates at least three cytoplasmic signaling pathways: the (1) Ras-Raf-mitogen-activated protein kinase (Ras-Raf-MAPK), (2) phosphatidylinositol 3-kinase (PI-3K)–protein kinase B (Akt), and (3) phospholipase C-gamma-1 (PLC-γ1) signaling pathways. The activation of PLC-γ1 is especially important for synaptic plasticity. PLC-γ1 activation leads to the production of IP3 and DAG, and the subsequent activation of protein kinase pathways [12,128,133,139]. The activation of protein kinase pathways by postsynaptic TrkB receptors results in the phosphorylation of NR1 and, most importantly, the NR2B subunits of NMDA receptors, leading to the potentiation of NMDA receptors and the long-term facilitation of synaptic currents in postsynaptic neurons [12,129,149,150,151,152]. In addition, TrkB receptor activation reduces potassium-chloride cotransporter 2 (KCC2) expression in the somato-dendritic membrane compartment of postsynaptic neurons [129,153]. KCC2 is a critical molecular factor in the maintenance of low intracellular chloride concentrations on which the hyperpolarizing effects of GABA_A_ and glycine receptors are based [154]. Thus, due to TrkB receptor-evoked downregulation of KCC2 expression and the subsequent increase in the intracellular chloride concentration, GABAergic and glycinergic inputs may trigger chloride ion outflux through GABA_A_ and glycine receptors, reducing postsynaptic hyperpolarization and facilitating network activities in dorsal horn nociceptive neuronal assemblies [12,155,156,157]. Acting on presynaptic axon terminals, TrkB receptor activation enhances the release of neurotransmitter substances stored in both clear and dense-core synaptic vesicles. This results in the amplification of glutamate release into the synaptic cleft [12,143] and establishes an autocrine loop that accounts for the BDNF-induced release of additional BDNF [143]. Because BDNF is costored with CGRP and SP in dense-core vesicles, presynaptic TrkB receptor activation has been shown to evoke CGRP and SP release, together with BDNF [143,147].

In addition to primary afferents, another source of BDNF has been identified in the spinal dorsal horn. In addition to glutamate, neuropeptides and BDNF, the axon terminals of nociceptive primary afferents also release cytokines, such as colony-stimulating factor (CSF-1), and chemokines, such as CCL-21 or CX3CL1 [67,139,158,159,160,161]. These substances activate microglia through their specific receptors. Activated microglia release biologically active substances including BDNF, which strengthens the effects of BDNF released from primary afferents [7]. Surprisingly, authors studying the biological impacts of microglia-derived BDNF reported that the effects were sex-dependent. The BDNF-dependent downregulation of KCC2 expression and the phosphorylation of NR2B subunits of NMDA receptors have been observed in male but not in female rats [162]. Although these findings may need further verification, they suggest that, in addition to neuron–neuron interactions, bidirectional communication between neurons and glial cells may substantially participate in the modulation of neural activities in laminae I–II of the spinal dorsal horn.

## 4. Presynaptic Modulation of Neurotransmitter Release from the Axon Terminals of Primary Afferents

Synaptic transmission from nociceptive primary afferents to spinal neurons is strongly regulated by a wide range of presynaptic receptors (Table 1). The expression patterns of these receptors seem to be highly variable, and their colocalization in functionally different subsets of nociceptive primary afferents, as well as in different functional states of the spinal nociceptive neural circuits, needs further verification. It seems to be increasingly accepted, however, that they rarely, if ever, aggregate in postsynaptic-membrane-like structures, and they are typically scattered along the cell membrane. Thus, the presynaptic modulation of primary nociceptive transmission typically follows the rules of volume transmission.

### 4.1. Presynaptic Inhibition via Metabotropic Glutamate Receptors

As discussed earlier, members of all three types of metabotropic glutamate receptors have been identified in laminae I–II of the spinal dorsal horn, which may activate signaling pathways that inhibit neurotransmitter release from the presynaptic axon terminals of nociceptive primary afferents.

#### 4.1.1. mGluR4 and mGluR7

These are presynaptic receptors inserted into the presynaptic membrane made by primary afferents. Both receptors are coupled to G_i/o_ proteins and may function as “rate limiting” autoreceptors decreasing the probability of glutamate release from the axon terminals [1,23,44,45,46]. This autoreceptor mechanism, however, can be functional only in a proportion of synapses, because mGluR4 and mGluR7 expression has been revealed only in ill-defined subsets of nociceptive primary afferents [51].

#### 4.1.2. mGluR2 and mGluR3

These are presynaptic receptors located at a considerable distance from the synaptic apposition [1,23]. Because of the very effective, high-affinity glial and neuronal glutamate uptake mechanisms, it is likely that synaptically released glutamate cannot spill out of the synaptic cleft into the extracellular space. Thus, these receptors presumably respond to the glutamate released by astrocytes adjacent to synaptic appositions [50]. Like mGluR4 and mGluR7, mGluR2 and mGluR3 are also coupled to G_i/o_ protein; thus, neurotransmitter release from the axon terminals can be further attenuated by their activation.

#### 4.1.3. mGluR5

mGluR5 is a postsynaptic receptor that is localized to the very peripheral zone of the postsynaptic membrane, establishing a so-called perisynaptic location [41,42]. This specific localization suggests that it can be activated only by an extensive release of glutamate from the presynaptic axon terminal owing to the repetitive stimulation of nociceptive primary afferents. In such cases, after the saturation of AMPA and NMDA receptors within the postsynaptic membrane, excess glutamate can flow to the perisynaptic area of the synaptic cleft. Although its activation is always associated with strong synaptic activity, mGluR5-G_q/11_ protein-evoked intracellular molecular events do not contribute to the generation of postsynaptic potentials, but rather activate a retrograde endocannabinoid signaling mechanism that leads to the attenuation of glutamate release from the presynaptic axon terminal [163,164,165,166].

#### 4.1.4. Retrograde Endocannabinoid Signaling

The retrograde endocannabinoid system at synapses between nociceptive primary afferents and secondary sensory neurons comprises type 1 cannabinoid receptor (CB1R), its endogenous ligand, 2-arachidonoyl glycerol (2-AG), a chemical apparatus responsible for the synthesis of 2-AG in postsynaptic dendrites, and another chemical apparatus that degrades 2-AG in presynaptic axons. Although the endocannabinoid system in the central nervous system, including the spinal cord, is more diverse than the molecular apparatus described above [164,167], this review focuses exclusively on events that occur at synapses made by nociceptive primary afferents in laminae I–II of the spinal dorsal horn.

In general, in the case of repetitive stimulation, presynaptic axons release enough glutamate to activate mGluR5 receptors in a perisynaptic location. Owing to the strong activation of NMDA receptors, the intracellular Ca^2+^ concentration of postsynaptic dendrites simultaneously increases. mGluR5-G_q/11_ protein complexes, together with the increased levels of cytoplasmic Ca^2+^, activate phospholipase C-β (PLC-β). Activated PLC-β catalyzes the cleavage of an integral lipid molecule of the cell membrane, phosphatidylinositol 4.5-biphosphate (PIP2), into IP3 and DAG. Finally, diacylglycerol lipase alpha (DGL-α), which is also inserted into the perisynaptic membrane adjacent to mGluR5, converts DAG to 2-AG. Lipophilic 2-AG, according to a poorly understood mechanism, is liberated from the cell membrane and diffuses out into the extracellular space to a distance of at least 20 µm [168]. The entire process is regarded as calcium-assisted receptor-driven endocannabinoid release [164]. The released 2-AG can then bind to CB1Rs expressed by the extrasynaptic membrane compartment of the presynaptic axon terminals. CB1Rs are coupled to G_i/o_ proteins, the activation of which inactivates AC, and most importantly blocks voltage-sensitive calcium channels [166], resulting in a short-term depression (STD) of neurotransmitter release, otherwise known as depolarization-induced suppression of excitation (DSE) [164,165,167].

Immunohistochemical staining and observations using conventional light and high-resolution electron microscopy clearly revealed that CB1Rs are expressed by nearly half of the CGRP-positive peptidergic nociceptive primary afferents and more than one-fifth of IB4-binding nonpeptidergic nociceptive primary afferents [169], whereas DGL-α is located at the perisynaptic membrane compartment of postsynaptic dendrites [170,171]. In addition to presynaptic axon terminals, released 2-AG may also act on CB1Rs expressed by astrocytes located close to 2-AG-releasing synapses. Astrocytes respond to the activation of their CB1Rs by releasing 2-AG (endocannabinoid-induced endocannabinoid release [8]), and 2-AG of astrocytic origin may also diffuse out and act on CB1Rs expressed on presynaptic axon terminals. Thus, short-term synaptic plasticity may lead to a long-term depression (LTD) of neurotransmitter release [164,167,172]. In summary, the retrograde endocannabinoid signaling mechanism can be regarded as the short- and long-term negative feedback regulation of neurotransmitter release from nociceptive axon terminals, the strength and degree of which may vary across a wide range of pain conditions evoked by the stimulation of various subgroups of nociceptive primary afferents.

Retrograde endocannabinoid signaling ends with the degradation of 2-AG in presynaptic axon terminals. This chemical process can be conducted via hydrolytic and oxidative pathways, and presumably both pathways are functional in the axon terminals of nociceptive primary afferents. The hydrolytic pathway is catalyzed primarily by monoacylglycerol lipase (MGL) [163]. Because MGL is expressed only in a proportion of CB1R-positive axon terminals [173], the oxidative pathway mediated by cyclooxygenase-2 (COX-2) and lipoxygenase (LOX) may also contribute to the degradation of 2-AG in nociceptive axon terminals.

### 4.2. Roles of Neuropeptides and Neurotrophins in Presynaptic Modulation

Neuropeptides and neurotrophins, their presynaptic receptors, and the effects of the activation of presynaptic receptors on synaptic plasticity have been discussed previously in detail. Here, only a short overview of these topics is given. Neuropeptides and neurotrophins may play major roles in the presynaptic modulation of neurotransmitter release from the axon terminals of primary afferents. Because they exert their effects via volume transmission, in addition to possible “homosynaptic” modulation, one must also consider a substantial “heterosynaptic” impact on the actual functional states of a much wider population of axon terminals (similar to retrograde endocannabinoid signaling), which may result in an unanticipated degree of enhancement or attenuation of neurotransmitter release. In addition, different neuropeptides and neurotrophins are commonly observed to be costored in LGVs [58,111,143,147]. Thus, these proteins are likely released together and act on their presynaptic receptors simultaneously, modulating each other’s effects on synaptic plasticity and moderating synaptic activities at the level of neuronal assemblies in a collaborative, and presumably rather complex, way.

#### 4.2.1. CGRP

The most widespread effects can be exerted by presynaptic CGRP receptors. Unfortunately, because of the lack of well-characterized antibodies, the presynaptic localization of these receptors is still poorly defined. According to the available indirect experimental data, however, both CLR–RAMP1 and CTR-RAMP1 receptors are likely expressed by virtually all the axon terminals of CGRP-expressing peptidergic primary afferents, and their activation may lead to increased neurotransmitter release from presynaptic axon terminals [4,5,10,62].

#### 4.2.2. SP

There is general agreement that NK receptors are not expressed by axon terminals. However, the phosphorylation and sensitization of NMDA receptor subunits by intracellular kinases activated by NK1 and NK3 receptors in postsynaptic neurons substantially increase the intracellular Ca^2+^ concentration. An increased level of Ca^2+^ may lead to the synthesis of PGE2, which may diffuse from postsynaptic neurons and bind to presynaptic EPRs, facilitating neurotransmitter release from presynaptic axons [5].

#### 4.2.3. GAL

The activation of GalR2 presynaptic receptors expressed by axon terminals of peptidergic primary afferents inactivates AC but elevates PLC levels. Among other metabolic effects, elevated PLC levels lead to an increase in the intraaxonal Ca^2+^ concentration, which facilitates glutamate and presumably also CGRP and SP release from presynaptic axon terminals [118]. Other reports have shown that nociceptive primary afferents may also express the GalR1 receptor [110,113,114,115]. However, whether presynaptic GalR1 receptors are functional and expressed on the same or different axon terminals where GalR2 receptors are located remains to be experimentally verified. If presynaptic GalR1 receptors are functional and expressed together with GalR2 receptors, the presynaptic action of GAL may be rather complex because GalR1 receptors are coupled to Gi/o proteins, which initiate signaling mechanisms leading to the hyperpolarization of the cell membrane and the subsequent depression of transmitter release [110,115].

#### 4.2.4. NPY

Presynaptic Y2 receptors are coupled to Gi/o proteins, which initiates metabotropic events including the activation of inward-rectifier K^+^ channels and the inhibition of Ca^2+^ channels in the membrane of presynaptic axons [11,120,121]. For this reason, Y2 receptors are thought to hyperpolarize the presynaptic axon terminal and reduce neurotransmitter release from both peptidergic and nonpeptidergic nociceptive primary afferents [11,120,124].

#### 4.2.5. BDNF

TrkB receptors are expressed in approximately one-third of nociceptive axon terminals in laminae I–II, and they are localized in both peptidergic and nonpeptidergic axon terminals [148]. Their activation drives multiple signaling pathways, resulting in the increased release of glutamate [12,143]. In addition to glutamate, TrkB receptor activation establishes a positive feedback loop for BDNF release. Upon binding BDNF, presynaptic TrkB receptors also initiate the release of BDNF [143]. In addition, because BDNF can be costored with CGRP and SP in the same dense-core vesicles, the activation of TrkB receptors may also facilitate the corelease of CGRP and SP [143,147].

### 4.3. Presynaptic Modulation of Synaptic Transmission by Gamma Aminobutyric Acid and Glycine

Through the release of glutamate, neuropeptides and neurotrophins from the axon terminals of nociceptive C and Aδ fibers and from nonnociceptive Aβ fibers, primary afferents activate a variety of neurons in the superficial spinal dorsal horn, including inhibitory neurons. The axons of some of these inhibitory neurons project back to the C and Aδ nociceptive primary afferent axon terminals, form axo-axonic contacts with them, and release gamma amino butyric acid (GABA) and/or glycine [13,174,175]. These axo-axonic contacts are routinely called axo-axonic synapses. However, this interpretation has not been substantiated by experimental data. That is, gephyrin, a postsynaptic scaffolding protein that is responsible for the clustering of GABA_A_ and glycine receptors in the postsynaptic membrane [176,177], is not present in the terminals of nociceptive primary afferents [178]. In the absence of gephyrin, GABA_A_ and glycine receptors cannot assemble into postsynaptic membranes. Most likely, they are loosely scattered within the cell membrane of nociceptive axon terminals. Consequently, these axo-axonic contacts should be named close appositions or “en passant boutons” [179]. Thus, the released GABA and glycine exert their effects on nociceptive primary afferent axon terminals via volume transmission rather than synaptic transmission.

#### 4.3.1. GABA

GABA is the most common inhibitory amino acid neurotransmitter in the central nervous system, and it acts on both ionotropic (GABA_A_) and metabotropic (GABA_B_) receptors [180]. Both types of receptors are expressed on both peptidergic and nonpeptidergic C- and Aδ-type nociceptive primary afferent terminals.

##### GABA_A_ Receptors

GABA_A_ receptors are pentameric chloride ion channels comprising two α, two β and one γ subunit in various combinations [181]. Thorough immunohistochemical studies utilizing highly specific antibodies against the different α, β, and γ subunits revealed that GABA_A_ receptors on the axon terminals of nociceptive primary afferents are constructed by only the α2, α3, β3 and γ2 subunits in laminae I–II of the spinal dorsal horn [156,182,183,184].

The activation of GABA_A_ receptors on nociceptive primary afferents can lead to presynaptic inhibition [13,175,179], but the molecular mechanism of this inhibition seems to differ between peptidergic and nonpeptidergic nociceptive primary afferents. That is, the intraaxonal chloride concentrations of these two sets of primary afferents are likely dissimilar. Together with other factors, the intracellular concentration of chloride in primary sensory neurons is regulated by two cation–chloride cotransporters—K^+^/Cl^−^ cotransporter 2 (KCC2) and Na^+^/K^+^ 2Cl^−^ cotransporter 1 (NKCC1). KCC2 extrudes Cl^−^ from cells, whereas NKCC1 transports Cl^−^ into the cytoplasm [185]. KCC2 expression is negligible and immunohistochemically undetectable in axon terminals within laminae I–II [154]. However, while NKCC1 is highly expressed in virtually all (approximately 95%) peptidergic (CGRP immunoreactive) nociceptive axon terminals, it is not expressed in nonpeptidergic (IB4-binding) axon terminals [186]. Thus, the intraaxonal Cl^−^ concentration is likely high in the axon terminals of peptidergic afferents and much lower in nonpeptidergic nociceptive primary afferents. For this reason, the activation of GABA_A_ receptors may cause an influx of Cl^−^ into the axon terminals and a consecutive hyperpolarization (reduction in the probability of neurotransmitter release) of the nonpeptidergic nociceptive primary afferents. However, owing to the high intraaxonal Cl^−^ concentration and the consecutive high chloride equilibrium potential (−30 mV in DRG neurons) [180,187], the opening of GABA_A_ receptor channels on the axon terminals of peptidergic nociceptive primary afferents induces Cl^−^ efflux and cell membrane depolarization. This well-known phenomenon, called primary afferent depolarization (PAD), that is due to various physiological and molecular mechanisms, including the inactivation of voltage-gated sodium and calcium ion channels and the attenuation of the amplitude of action potentials propagated by primary afferents, may also result in the presynaptic inhibition of transmitter release [13,175,179,188]. In the case of increased NKCC1 expression, however, which has been demonstrated in DRG neurons [156,187,189,190] and in the spinal dorsal horn [191] (Galan and Cervero, 2005) in chronic pain models, owing to the even greater increase in intraaxonal Cl^−^ concentration, the augmentation of PAD may generate action potentials in primary afferents, thus inducing glutamate release and activating spinal neurons [192,193].

##### GABA_B_ Receptors

GABA_B_ receptors are metabotropic receptors composed of two subunits—GABA_B1_ and GABA_B2_. The GABA_B1_ subunit can bind GABA but cannot bind to G proteins, whereas the GABA_B2_ subunit cannot bind GABA but can be coupled to G proteins, making the membrane expression of the receptor complex possible [194]. There are two isoforms of the GABA_B1_ subunit, GABA_B1a_ and GABA_B1b_, but more than 90% of DRG neurons give rise to C and Aδ primary afferents, which express GABA_B1a_ mRNA [195]. Thus, most of the functional GABA_B_ receptors expressed in the spinal axon terminals of C and Aδ nociceptive primary afferents are formed as GABA_B1a_–GABA_B2_ complexes. Unfortunately, there are still no data in the literature concerning how many peptidergic and nonpeptidergic nociceptive axon terminals express GABA_B_ receptors on their cell membrane.

GABA_B_ receptors are coupled to G_i/o_ proteins, the activation of which inhibits AC and voltage-gated Ca^2+^ channels, and activates inward-rectifier K^+^ channels, subsequently attenuating neurotransmitter release from the axon terminals [180,196,197]. Thus, the activation of GABA_B_ receptors strongly inhibits neural transmission from nociceptive primary afferents to secondary sensory neurons in the spinal dorsal horn.

#### 4.3.2. Glycine and Glycine Receptors

Although glycine has been historically regarded as a cotransmitter in a proportion of GABAergic neurons, an abundance of glycine-only (glycine transporter 2 positive and glutamic acid decarboxylase negative) axon terminals in the superficial spinal dorsal horn have recently been identified [198]. In addition, the importance of glycine-only synaptic inhibition in spinal nociceptive information processing has also been confirmed [199,200]. The effect of glycine is mediated by glycine receptors (GlyRs), which are pentameric chloride-conducting ion channels. There are five cloned subunits of the channel, α1-4 and β. The different α subunits can be associated with each other to form homomeric receptors, or with the β subunit to form heteromeric receptors. Only the β subunit can interact with the postsynaptic scaffolding protein gephyrin; thus, it is required for the formation of receptor clusters in the postsynaptic membrane [201]. Because gephyrin is missing from the axon terminals in the superficial spinal dorsal horn, the β subunit is not required for the formation of presynaptic GlyRs in this region. Thus, presynaptic GlyRs expressed by the axon terminals of nociceptive primary afferents are supposedly constructed only by α subunits, preferentially by the α1 and α3 subunits [201].

The abundance of GlyRs on the axon terminals of nociceptive primary afferents is limited. Many authors claim that they are absent from the axon terminals of primary sensory neurons [95,178]. Recently, however, Miranda et al. [202] reported that glycinergic (glycine transporter 2 immunoreactive) axon terminals make close appositions with a subset of nonpeptidergic (IB4-binding) nociceptive C-fiber axon terminals. In addition, Bae et al. [203] reported that trigeminal sensory neurons and their central axon terminals were immunoreactive for the α3 subunit. It has also been shown that the axon terminals positively stained for the α3 subunit contained clear round, but not dense-core, synaptic vesicles, characteristic of nonpeptidergic axon terminals [203]. These findings indicate that the glycinergic presynaptic inhibition of nociceptive primary afferents is inferior to GABAergic presynaptic inhibition. Its contribution to the generation of PAD is negligible; glycine-mediated PAD cannot be recorded by traditional methods [179], but glycinergic presynaptic inhibition may play a role in targeting a subpopulation of nonpeptidergic nociceptive axon terminals.

### 4.4. Presynaptic Inhibition of Synaptic Transmission by Endogenous Opioid Peptides

Opioids have long been known to modulate pain-related information processing in the spinal dorsal horn. All three “typical” endogenous opioid peptides, enkephalin, dynorphin and endorphin, have been detected in the superficial spinal dorsal horn [204,205]. Independent of the lengths of their amino acid chains, the first four amino acids are identical in the three peptides (Tyr-Gly-Gly-Phe), and the fifth amino acid can be either methionine (met-enkephalin and endorphin) or leucin (leu-enkephalin and dynorphin) [205]. In addition to “typical” endogenous opioids, a fourth opioid peptide, named endomorphin, has also been identified in the central nervous system [170,206]. Endomorphin is a tetrapeptide present in two isoforms, endomorphin-1 and endomorphin-2. The two isoforms show distinct patterns of distribution. Endomorphin-1 is widely distributed in the brain and upper brain stem, whereas endomorphin-2 has been found in the lower brain stem, spinal cord and dorsal root ganglia [206,207].

In the superficial spinal dorsal horn, endogenous opioids are released by different cell populations. (1) Transgenic, morphological and electrophysiological studies revealed that enkephalins and dynorphins are synthesized and released almost exclusively by intrinsic excitatory and inhibitory interneurons, which may or may not receive direct innervation from nociceptive primary afferents [208,209,210,211,212]. To a minimal extent, descending fibers of brainstem origin may also contribute to the enkephalinergic innervation of the spinal dorsal horn [210]. Interestingly, all dynorphin-positive inhibitory interneurons express galanin and carry sst2A receptors [208,213]. The axons of enkephalin- and dynorphin-containing neurons make close contacts, but not synapses, with the axon terminals of nociceptive primary afferents [210]. (2) β-Endorphin in laminae I–II is presumably secreted only by microglia under the control of interleukin-10 (IL-10) [67,214,215,216,217]. The secreted β-endorphin may diffuse from the site of release and may act on various neuronal elements, including the axon terminals of nociceptive axon terminals. (3) Endomorphin-2 is synthesized primarily by small- and medium-sized DRG neurons, and is released by their axon terminals in the superficial spinal dorsal horn [206,218]. In addition to nociceptive axon terminals, some descending fibers arising from the nucleus of the solitary tract may also release endomorphin-2 in laminae I–II [219]. With respect to the DRG neurons and their central axon terminals, endomorphin-2 is localized primarily to peptidergic nociceptive primary afferents. Virtually all endomorphin-2-containing primary afferents express CGRP, and approximately two-thirds of them are also positive for SP [206,218,220]. Within these axon terminals, endomorphin-2 is costored with CGRP and SP in dense-core synaptic vesicles [170,218]; thus, it is likely that endomorphin-2 is released together with CGRP, and frequently also with SP, in laminae I–II. However, Nydahl et al. [170] reported that a limited number of IB4-binding nonpeptidergic nociceptive primary afferents showed endomorphin-2 immunoreactivity.

Early autoradiographic binding studies together with later sequence analysis and selective gene deletions confirmed three opioid receptors in the central nervous system: mu receptors (MORs), delta receptors (DORs) and kappa receptors (KORs) [221,222]. All of them are G_i/o_ protein-coupled metabotropic receptors showing remarkable homology in their transmembrane domains, intracellular loops and intracellular C-terminal tails. However, the extracellular loops and the extracellular N-terminal tails show major dissimilarities, which make the three receptors pharmacologically different from each other [223,224]. For this reason, the three receptors have different affinities for different endogenous opioid peptides. Endomorphins bind to MORs with high selectivity and affinity, 1000-fold more potently than to DORs [206,218,220,225]. β-Endorphin also preferentially binds to MORs, but can also bind to DORs, although with a 10-fold lower affinity [205,222,223]. Enkephalins activate MORs and DORs with similar effectiveness [209,222], whereas dynorphin is regarded as an almost selective ligand for KORs [205,222,223]. After binding their ligands, the three receptors initiate identical intracellular signaling via Gi/o protein coupling. Following their activation, opioid receptors inactivate AC and inhibit cAMP production. They block voltage-sensitive Ca^2+^ channels, suppress Ca^2+^ influx and presynaptically repress neurotransmitter release from axon terminals. In addition, they activate inward-rectifier K^+^ channels, thereby hyperpolarizing cell membranes and preventing action potential propagation [223,224,226].

In the spinal dorsal horn, opioid receptors are expressed in both pre- and postsynaptic positions. All types of opioid receptors are expressed by nociceptive DRG neurons and their axon terminals in laminae I–II [221,222], but the levels of expression of the different receptors in the spinal axon terminals are very different. Approximately 75%, 20% and 5% of MORs, DORs and KORs expression, respectively, were lost after dorsal rhizotomy [227]. Presumably because of the low level of expression of KOR, investigations of dynorphin-KOR-mediated presynaptic events in nociceptive primary afferents have yielded contradictory and inconclusive data, or simply negative results in most cases. This might be the reason why most recent experiments investigating the presynaptic distribution and actions of opioid receptors on nociceptive primary sensory axon terminals have focused on MORs and DORs, whereas KORs were simply neglected in these studies. Owing to the lack of reliable data concerning the expression of KORs in the axon terminals of nociceptive primary afferents, this review provides an overview of only the expression of MORs and DORs in nociceptive axon terminals in laminae I–II.

In contrast to early reports, it has recently become well accepted that MOR and DOR expression is mostly, if not completely, distinct in DRG neurons and on the axon terminals of nociceptive primary afferents [95,220,228,229,230]. It has also been shown that the two receptors are internalized and function independently [230]. On the basis of experimental data obtained from in situ hybridization, single-cell RNA sequencing, immunohistochemistry, transgenic experiments and electrophysiology, it is increasingly accepted that MOR is expressed in C- and Aδ-type, NGF-dependent peptidergic nociceptive primary afferents, whereas DOR is restricted to the axon terminals of C-type, IB4-binding nonpeptidergic nociceptive primary afferents in laminae I–II [95,220,228,230].

Concerning the remarkable colocalization of endomorphin and MORs in the axon terminals of peptidergic nociceptive primary afferents, it is likely that endomorphin can primarily be regarded as an autoregulatory factor regulating synaptic transmission as an inhibitory feedback mechanism at the peptidergic C and Aδ primary afferents in laminae I–II [206,220,225,231]. In contrast to that of endomorphin, the action of enkephalin on nociceptive axon terminals is indirect. In this case, first, the enkephalin-releasing interneurons in laminae I–II must be activated by incoming nociceptive signals. The axons of these interneurons may release encephalin, which can act on both presynaptic MORs and DORs, negatively modulating the activities of both peptidergic and nonpeptidergic nociceptive primary afferents [209]. β-Endorphins may also enhance the activation of presynaptic MORs and DORs to a certain degree, but this enhancement requires the activation of microglia and the production of IL-10 [67,214,215,216,217].

### 4.5. Presynaptic Inhibition of Synaptic Transmission by the Nociception/Orphanin FQ Peptide and Receptor

In 1994, a new Gi/o protein-coupled metabotropic receptor that presented more than 60% sequence homology with opioid receptors was discovered. For this reason, it was named opioid-related receptor (ORL1) [232]. One year later, two independent groups identified its endogenous ligand, a 17-amino-acid-long peptide, which shows close similarity to dynorphin A. One of the groups named the new peptide nociceptin, and the other named it orphanin FQ [233]. Despite their close similarity with opioid peptides and receptors, the new receptor showed very low affinity for “classical” opioid peptides, and the newly discovered ligand bound to “classical” opioid receptors with very low affinities [234,235,236,237,238,239]. To distinguish the new peptide and receptor clearly from other opioid ligands and receptors, they were finally named the nociception/orphanin FQ (N/OFQ) peptide and N/OFQ peptide (NOP) receptor [240]. At the cellular level, the activation of the NOP receptor by the N/OFQ peptide initiates intracellular events such as the activation of “classical” opioids and other G_i/o_ protein-coupled metabotropic receptors. It inactivates AC, decreases the level of cAMP, blocks voltage-gated Ca^2+^ channels and activates inward-rectifier K^+^ channels [235,237,241,242]. For some reason, however, the systemic activation of NOP receptors, especially in nonhuman primates, induces analgesia without the unwanted side effects associated with MOR activation, such as drug dependence, respiratory depression or constipation [234,241,242]. For this reason, N/OFQ peptide-NOP receptor signaling has recently attracted increasing interest.

Both N/OFQ peptide and NOP receptors are widely distributed in the peripheral and central nervous systems, including the spinal cord and DRG [235,238,243,244,245,246,247]. Owing to the lack of well-characterized and reliable antibodies against the N/OFQ peptide, our present knowledge about the localization of the N/OFQ protein in the spinal cord and DRG is based only on early in situ hybridization studies. These studies revealed that N/OFQ and preproN/OFQ mRNAs were both present in neurons in the DRG and superficial spinal dorsal horn, primarily in laminae II [242,248,249]. Thus, the N/OFQ peptide might be released in the superficial spinal dorsal horn, mostly by lamina II interneurons, with some contribution from the axon terminals of nociceptive primary afferents. On the other hand, autoradiographic analysis revealed that rhizotomy reduced [^3^H] nociceptin (N/OFQ) binding in the dorsal horn ipsilateral to a deafferentation by approximately 18% [250], indicating that NOP receptors are expressed primarily by interneurons, but also by axon terminals of nociceptive primary afferents in laminae I–II.

In nociceptive primary afferents, a more recent transgenic approach, reporter fluorescent protein expression (NOP-eGFP), revealed a more detailed localization pattern of NOP receptors in the mouse superficial spinal dorsal horn and DRG [245]. NOP-eGFP labeling was detected in both small and large DRG neurons. One-third of the NOP-eGFP-positive DRG neurons coexpressed CGRP, and one-fifth of them bound IB4. Intense NOP-eGFP staining was also observed in laminae I–II of the spinal dorsal horn, where CGRP-positive and IB4-binding primary afferents terminate [245], suggesting that a population of both peptidergic and nonpeptidergic nociceptive primary afferents carry presynaptic NOP receptors on their spinal axon terminals. Thus, one may assume that the N/OFQ peptide released by primary afferents may not only inhibit spinal interneurons, but may also act as an autoreceptor, inhibiting neurotransmitter release from a presently undefined population of primary afferents.

The expression levels of MOR are similar to those of CGRP in NOP-eGFP positive DRG neurons [245]. In addition, as mentioned earlier, at least one population of IB4-binding nonpeptidergic nociceptive primary afferents in laminae I–II are positive for DOR [59,95,220,228]. These findings suggest that NOP receptors might collaborate with MORs and DORs on the axon terminals of peptidergic and nonpeptidergic nociceptive primary afferents in laminae I–II, respectively. This notion is reinforced by an electrophysiological study demonstrating that a substantial proportion of small peptidergic DRG neurons are responsive to both N/OFQ and DAMGO [251].

### 4.6. Presynaptic Facilitation of Synaptic Transmission by the Purinergic System

The purine nucleotide adenosine 5′-triphosphate (ATP) can be found in all living cells, and is known as a molecule that is indispensable for intracellular energy transfer. In addition to this vital role in cell biology, however, ATP is also used as a transmitter in the nervous system. Cytosolic ATP in axon terminals can be transported into clear synaptic vesicles by vesicular nucleotide transporter (VNUT) [252,253]. ATP-containing vesicles can fuse with the plasma membrane of axon terminals, and release ATP into the synaptic cleft and the extracellular space in a Ca^2+^-dependent manner [254,255]. It was suggested that ATP can be co-stored with glutamate in the same synaptic vesicles [256], but convincing experimental results have demonstrated that ATP is stored in a distinct pool of synaptic vesicles, and only presynaptic action potentials synchronise the release of glutamate and ATP from axon terminals [257,258]. In addition to vesicular transport, ATP can be transported from the cytoplasm to the extracellular space by specific transport mechanisms, connexin and pannexin hemichannels, and voltage-dependent anion channels [259], but it is likely that these additional mechanisms contribute to the release of ATP to a minimal extent (if at all) in the central nervous system [253].

Early physiological findings suggested that spinal axon terminals of nociceptive primary afferents may release ATP [260,261]. However, VNUT, which seems to be the only molecule that can transport ATP into synaptic vesicles, has never been detected in the central axon terminals of nociceptive primary afferents, although it is expressed in DRG neurons [262]. Because of this ambiguity of the recently available experimental data, it cannot be stated for sure that ATP can be released from the spinal axon terminals of nociceptive primary afferents; this requires further experimental verifications. There is strong evidence, however, that a wide range of neurons in the superficial spinal dorsal horn express VNUT, and in case of repetitive stimulation, the VNUT-positive neurons can discharge a substantial amount of ATP into the extracellular space [253,263]. Astrocytes may also contribute to ATP release in some areas of the central nervous system [264], but it was shown that ATP of astrocytic origin has limited, if any, significance in spinal pain processing [253].

After its release from local interneurons, ATP diffuses in the extracellular space, while it undergoes enzymatic degradation by cell surface-associated and secreted ectonucleotides, providing a mechanism for the formation of adenosine diphosphate (ADP), adenosine monophosphate (AMP), and adenosine [254,255,259]. Thus, in the case of ATP discharge, not only ATP but also ADP and adenosine are present in the extracellular space, and are used as transmitters in the nervous system [265,266]. At the end of this enzymatic process, adenosine can be converted to hypoxanthine and/or removed from the extracellular space by adenosine membrane transporters [265]. Because of the simultaneous presence of the three purinergic transmitters, ATP, ADP and adenosine, in the extracellular space, and the extensive diffusion of these substances, the spatial–temporal properties of purinergic signaling can be very complex and highly dependent on the distribution of purinergic receptors and the local activities of ectonucleotides.

Purinergic receptors belong to two main families: P1 or A receptors and P2 receptors. P2 receptors are further subdivided into P2X and P2Y receptors. The specific ligand for P1/A receptors is adenosine. Both subtypes of P2 receptors can be activated by ATP, and P2Y receptors can bind also ADP [265,267]. P2X receptors are ligand-gated ion channels assembled from three protein subunits and permeable to Na^+^, K^+^ and Ca^2+^ ions [266]. Seven P2X receptor subunits have been identified (P2X 1–7), which can form both homo- and hetero-multimers creating the trimeric subunit structure of the receptor channel. So far, six homomeric and six heteromeric P2X receptors have been discovered [259,265,266,268,269]. There is general agreement that there are eight P2Y receptors (P2Y_1,2,4,6,11,12,13,14_). All are G-protein coupled receptors; P2Y_1,2,4,6,11_ subtypes are coupled to G_q/11_ protein, whereas P2Y_12,13,14_ subtypes are coupled to G_i/o_ protein [265,266,268,269]. Four adenosine (P1/A) receptors have been cloned and characterized, A_1,2A,2B,3_. All are G protein-coupled receptors; A_1_ and A_3_ receptors are coupled to G_i/o_, whereas A_2A_ and A_2B_ receptors are coupled to G_s_ protein [265,266].

It has been convincingly demonstrated that P2X_3_ homomeric and P2X_2/3_ heteromeric receptors are heavily expressed in IB4-binding nociceptive non-peptidergic axon terminals in lamina IIi [129,232,267,269,270,271,272]. Based on their expression in IB4-binding and CGRP containing DRG neurons, it has been suggested that A_1_, P2Y_1_ and P2X_3_, P2Y_2_ and P2Y_4_ receptors can also be expressed in non-peptidergic and peptidergic axon terminals [260,261,273], respectively, but the expressions of these receptors in the spinal axon terminals of nociceptive primary afferents have not been demonstrated. Thus, based on currently available experimental data, one may conclude that extracellular ATP can increase glutamate release from non-peptidergic, but not from peptidergic nociceptive, primary afferents in the superficial spinal dorsal horn via the activation of Ca^2+^-permeable presynaptic P2X_3_ and P2X_2/3_ ionotropic receptors.

The functional significance of presynaptic P2X_3_ and P2X_2/3_ receptors in the enhancement of glutamate release from non-peptidergic nociceptive primary afferents is well established. It is likely, however, that the most fundamental aspect of purinergic signaling in spinal pain processing is represented by a neuron–microglia–neuron communication system in which microglial purinergic receptors play inevitable roles. As mentioned earlier, in the case of their repetitive stimulation caused by peripheral nerve injury or inflammation, spinal axon terminals of nociceptive primary afferents release cytokines and chemokines [6,7,139,158,160,161,274]. Due to the activation of their receptors, cytokines and chemokines initiate fundamental changes in the morphology, motility, biochemical properties, and receptor expression patterns of microglia, which convert quiescent microglia into a new functional state called “activated or reactive microglia”. Concerning the purinergic system, activated microglia increase the expression of P2X_4_, P2X_7_ and P2Y_12_ receptors [267,268,269,275,276,277,278,279]. The activation of P2Y_12_ receptors by ATP and ADP induces membrane ruffling and influence the abilities of microglia to extend their processes, which may make microglia–neuron communications more efficient [277,278,280,281]. Owing to the activation of P2X_7_ receptors by ATP, microglia synthesize and release proinflammatory cytokines such as interleukin-1β (IL-1β) [281,282,283]. After its release, IL-1β diffuses in the extracellular space and binds to interleukin-1 receptor type 1 (IL-1R1) [284]. IL-1R1 is distributed along dendrites of neurons in the superficial spinal dorsal horn, and shows remarkable enrichments in postsynaptic membranes of glutamatergic synapses [285,286]. The activation of synaptic IL-1R1 amplifies NMDA receptor-mediated synaptic currents via the phosphorylation of NR1 and NR2B subunits of NMDA receptors [287].

Although P2X_4_ receptors are weakly expressed in quiescent microglia, their expression is strongly increased in activated microglia [275,279]. Owing to the activation of P2X_4_ receptors by ATP, activated microglia synthesizes and releases BDNF [267,269,279]. Because activated microglia invade both lamina I and lamina II in chronic pain conditions, both ATP and BDNF diffuse widely in the extracellular space, and TrkB receptors are expressed postsynaptically by a wide range of interneurons and presynaptically by both peptidergic and non-peptidergic nociceptive primary afferents, as a result of which the BDNF released by activated microglia exerts profound pre- and postsynaptic effects on the entire pain processing apparatus of the superficial spinal dorsal horn [267,269,279]. As discussed earlier, due to the activation of presynaptic TrkB receptors on both peptidergic and non-peptidergic axon terminals, microglia-derived BDNF enhances not only glutamate, but also CGRP and SP, release from the axon terminals of nociceptive primary afferents, contributing to the development of central sensitization, tactile allodynia and chronic pain [12,129,149,150,151,152,155].

### 4.7. Presynaptic Modulation of Synaptic Transmission by Monoaminergic Descending Fibers

Some supraspinal catecholaminergic (noradrenergic and dopaminergic) and serotoninergic nuclei project into the spinal cord, and all three types of monoaminergic descending systems modulate spinal nociceptive, pain-related information processing, including synaptic transmission from nociceptive primary afferents to spinal interneurons through presynaptic mechanisms [9,288,289]. After their synthesis, all types of monoamines are transported into small clear synaptic vesicles by the H^+^-coupled vesicular monoamine transporter (VMAT), which is present in two isoforms, VMAT1 and VMAT2 [290]. The expression patterns of the two isoforms show striking differences. Although some species-dependent variations have been observed, VMAT2 is expressed primarily in neurons, whereas VMAT1 is primarily expressed in endocrine cells [290]. Because monoamines are stored in synaptic vesicles, they exit from the axon terminals via vesicular release at both synaptic and nonsynaptic appositions. In the spinal dorsal horn, the descending monoaminergic fibers form axodendritic and axosomatic synapses [291,292], but axoaxonic synapses established by the spinal terminals of the descending monoaminergic axons have never been identified. Thus, one may assume that the effects of monoamines on the axon terminals of nociceptive primary afferents are exerted according to nonsynaptic volume transmission. The duration of the postsynaptic effects of monoamines, among other factors, is regulated by monoamine transporters (MATs), which remove monoamines from the extracellular space [293]. Although all monoamines are transported into synaptic vesicles by one vesicular transporter (VMAT), reuptake is performed by MATs specific for individual monoamines. Thus, there are three MATs, dopamine transporter (DAT), serotonin transporter (SERT) and noradrenaline transporter (NET), all of which are widely expressed in the spinal dorsal horn [293].

#### 4.7.1. Noradrenaline

Catecholamines are biosynthesized from the amino acid tyrosine. Along this biosynthetic pathway, tyrosine is first converted to DOPA by tyrosine hydroxylase, which is transformed to dopamine via decarboxylation. In certain neurons, which are referred to as dopaminergic neurons, this is the end of the biosynthetic process. In other neurons, dopamine is further converted to noradrenaline by dopamine-beta-hydroxylase. Noradrenaline can be transformed to adrenaline via methylation, but this process occurs primarily in the peripheral nervous system. Although there are three small adrenergic cell groups in the brainstem, adrenergic neurotransmission does not play a significant role in spinal pain processing.

Noradrenergic neurons are confined to the caudal brainstem (medulla oblongata and pons) and are categorized into seven cell groups, which are referred to as A1–A7. The three pontine nuclei, A5, A6 (also known as the locus coeruleus) and A7, project into the spinal cord [289,294,295,296,297]. Most ponto-spinal noradrenergic projections (~80%) arise from the ventral part of the locus coeruleus [298,299].

There are three types of noradrenergic receptors, α1, α2, and β. All three types are metabotropic receptors, but the different receptors are coupled to different G proteins. The α1-, α2- and β-adrenoceptors are coupled to G_q/11_, G_i/o_ and G_s_ proteins, respectively. Thus, their activation initiates quite different cytoplasmic signaling mechanisms [294,295,296]. In the spinal dorsal horn, α1- and α2- adrenoceptors dominate, and both are widely distributed in laminae I–II. While both α1 and α2 adrenoceptors are presented in spinal interneurons, only the α2-adrenoceptor has been identified in the axon terminals of nociceptive primary afferents [289,295,297,300]. The α2 adrenoceptor has three isoforms, α2A, α2B, and α2C, in the central nervous system, but only the α2A isoform has been found in nociceptive primary afferents [289,297]. Moreover, the α2A adrenoceptors seem to be expressed exclusively by capsaicin-sensitive, SP- and CGRP-containing nociceptive axon terminals [300]. Because α2A adrenoceptors are coupled to G_i/o_ proteins, their activation results in the inactivation of AC and blockage of voltage-gated Ca^2+^ channels, as well as the activation of inward-rectifier K^+^ channels, and consequently, the suppression of neurotransmitter release from the SP-containing axon terminals of nociceptive primary afferents [295,296,297].

#### 4.7.2. Dopamine

Dopaminergic neurons are grouped into eight nuclei, defined as A8–A16, and are distributed from the mesencephalon to the telencephalon. From these nuclei, only the A11 hypothalamic region sends descending fibers to the spinal cord, mostly to the superficial dorsal horn, but some dopaminergic fibers also terminate in the ventral horn and in the gray matter surrounding the central canal [288,292,301,302,303].

Five dopaminergic receptors (D1–D5) have been detected in the nervous system. All of them are metabotropic receptors that are classified into two functional groups: D1-like receptors, composed of D1 and D5, and D2-like receptors, composed of D2, D3 and D4. D1-like receptors are expressed on dendrites, whereas D2-like receptors are expressed on both dendrites and axons [304]. D1-like receptors are coupled to G_s_ proteins, whereas D2-like receptors are coupled to G_i/o_ proteins. Thus, the two receptor families initiate antagonistic intracellular signaling mechanisms [305,306]. The activation of D1-like receptors activates, whereas the activation of D2-like receptors inactivates, AC. In addition, presynaptic D2-like receptor activation initiates intracellular signaling, which suppresses voltage-gated Ca^2+^ channels and potentiates inward-rectifier K^+^ channels, resulting in the depression of neurotransmitter release from axon terminals [305,306].

Radiolabeled binding studies and immunohistochemical labeling revealed the expression of D2-like receptors, primarily D2, but also D3 and D4, in the superficial spinal dorsal horn. mRNAs encoding D2, D3 and D4 receptors have also been detected in DRG neurons, suggesting that the axon terminals of nociceptive primary afferents in laminae I–II may express D2, D3 and D4 receptors [305]. This notion has been reinforced by a recent pharmacological experiment in which Lu and his collaborators reported that the application of dopamine inhibited nociceptive Aδ and C fiber inputs to lamina I projection neurons via presynaptic actions [9]. These results also suggest that the inhibitory effects were mediated by D3 and D4 receptors [9]. In addition, it was previously demonstrated that the intrathecal application of D2-like receptor agonists, but not D1-like receptor agonists, produced analgesic effects on mechanical nociception, and these effects could be abrogated by the application of D2, D3 and D4 receptor antagonists [288,307].

Moreover, several indirect experimental studies suggest that axon terminals of some nociceptive primary afferent populations may express D2-like receptors, but are devoid of D1-like receptors, and that the activation of D2-like receptors results in the strong presynaptic inhibition of nociceptive neurotransmission. These rather convincing indirect findings, however, need to be substantiated by direct high-resolution morphological studies, possibly combined with genetic tools, to clearly show the expressions of the various dopamine receptors on specific subpopulations of nociceptive primary afferents.

#### 4.7.3. Serotonin

Serotonin (5-hydroxytryptamine; 5-HT) is synthesized from the amino acid tryptophan via hydroxylation and subsequent decarboxylation. Serotoninergic neurons are confined to the raphe nuclei of the brainstem and are arranged in nine cell groups (B1–B9). Spinally descending serotonergic fibers arise from the B3 cell group, which corresponds to the rostral ventromedial medulla (RVM) [308,309,310]. Serotoninergic neurons in the RVM are distributed in the nucleus raphe magnus and adjacent reticular formation, including the lateral parvocellular reticular nucleus and the ventral portion of the nucleus gigantocellularis [311]. The descending raphe-spinal serotoninergic fibers terminate in various areas of the spinal cord. Those that terminate in the superficial spinal dorsal horn arise primarily from the lateral parvocellular reticular nucleus [308]. Some of the serotonergic axons form axodendritic and axosomatic synapses in laminae I–II, but most terminate with nonsynaptic varicosities, suggesting that the released serotonin acts mostly on the spinal neurons and axon terminals of primary afferents exclusively through volume transmission in laminae I–II [305,309].

Seven families of serotonin receptors (5-HT_1–7_), with 14 subtypes, have been identified in the mammalian central nervous system. Six of the seven 5-HT receptor families are metabotropic receptors, whereas the 5-HT_3_ receptor is ionotropic and permeable to Na^+^, K^+^ and Ca^2+^ ions. Thus, the activation of 5-HT_3_ receptors depolarizes the cell membrane. The different metabotropic 5-HT receptors show a high degree of homology in their transmembrane domains, but their terminal portions and intracellular loops present marked differences in their amino acid sequences. They are coupled to various G proteins; 5-HT_1_ and 5-HT_5_ receptors are coupled to G_i/o_ proteins, 5-HT_2_ receptors are coupled to G_q/11_ proteins, whereas 5-HT_4_, 5-HT_6_ and 5-HT_7_ receptors are coupled to G_s_ proteins [309,310,312,313]. The distribution of the different 5-HT receptors in the central nervous system shows several unique features, but the biological significance of this diversity still needs proper interpretation. In accordance with the suggested predominance of serotoninergic volume transmission, 5-HT receptors have been found to be strongly expressed in extrasynaptic membrane compartments of both dendrites and axon terminals [314,315].

With respect to the modulation of spinal nociceptive information processing, the contributions of 5-HT_1_, 5-HT_2_, 5-HT_3_ and 5-HT_7_ receptors have been identified [316,317], and in addition to spinal neurons, all these receptors are localized on the spinal axon terminals of nociceptive primary afferents. The literature concerning the expression patterns of the different 5-HT receptors on distinct types of nociceptive primary afferents is incomplete. The available data suggest that different 5-HT receptors are expressed only by a limited subpopulation of nociceptive primary afferents, and that their expression patterns may vary depending on the type of afferent.

##### 5-HT_1_ Receptors

The 5-HT_1_ receptor is considered the main type of 5-HT receptors in the spinal cord, and two subtypes, 5-HT_1A_ and 5-HT_1B_, appear to be present on the spinal axon terminals of nociceptive primary afferents [309,316,317]. Dorsal rhizotomy or neonatal treatment with capsaicin eliminating TRPV1-expressing neurons decreases both 5-HT_1A_ and 5-HT_1B_ radioligand binding in laminae I–II. In addition, 5-HT_1B_ mRNA has been detected in some DRG neurons [309]. The colocalization of 5-HT_1B_ receptors with both SP and CGRP has also been demonstrated [318,319], indicating that 5HT_1B_ receptors are expressed in a subpopulation of peptidergic nociceptive primary afferents.

As mentioned earlier, 5-HT_1_ receptors are coupled to Gi/o proteins. Thus, the activation of the 5HT_1A_ and 5-HT_1B_ receptors on different subsets of nociceptive primary afferents inactivates AC and decreases the level of cAMP. In addition, voltage-gated Ca^2+^ channels are also inhibited, whereas the probability of opening of inward-rectifier K^+^ channels is increased. Thus, through binding to 5HT_1A_ or 5HT_1B_ receptors, serotonin attenuates neurotransmitter release from a poorly defined subset of nociceptive primary afferents, and exerts an antinociceptive effect on spinal nociceptive information processing [309,310,312,313,315].

##### 5-HT_2_ Receptors

5-HT_2_ receptors are less abundant in the spinal dorsal horn than 5-HT_1_ receptors are, but the expressions of 5-HT_2A_ receptors on nociceptive primary afferents cannot be ignored [309,317]. Immunohistochemical studies demonstrated the expression of 5-HT_2A_ receptors on DRG neurons. In addition, smaller 5-HT_2A_-positive neurons often bind IB4, and some of them also express SP or TRPV1 [309,320]. Moreover, labeling for 5-HT_2A_ in axon terminals containing large granular vesicles has also been observed in the superficial spinal dorsal horn [321].

5-HT_2_ receptors are coupled to G_q/11_ proteins. Thus, after binding serotonin, 5-HT_2_ receptors activate PLC and degrade PIP3, leading to the activation of PKC and the production of IP3 and DAG [310,312,313]. This entire process may lead to the accumulation of cytoplasmic Ca^2+^ and membrane depolarization.

##### 5-HT_7_ Receptors

The superficial spinal dorsal horn presents a modest abundance of 5-HT_7_ receptors. 5-HT_7_ receptor mRNA has been detected in small-diameter DRG neurons and in unmyelinated and thinly myelinated primary afferent fibers, suggesting that it is expressed on the spinal axon terminals of both C and Aδ nociceptive primary afferents [305,320,322].

5-HT_7_ receptors are coupled to Gs proteins; thus, they can activate AC and increase cAMP production. This process enhances Ca^2+^ flux through L-type Ca^2+^ channels, which results in membrane depolarization [179,310,312,313].

##### 5-HT_3_ Receptors

Axon terminals immunoreactive for the 5-HT_3_ receptor are densely arranged in laminae I–II of the spinal dorsal horn. Quantitative morphological analysis revealed that most of them were axon terminals of local interneurons; only 10% and 3% of the axon terminals expressed CGRP and bound IB4, respectively [323], suggesting that undefined subpopulations of both peptidergic and nonpeptidergic nociceptive primary afferents express 5-HT_3_ receptors. In addition, other studies have shown that dorsal rhizotomy or neonatal capsaicin treatment decreases 5-HT_3_ ligand binding in the superficial spinal dorsal horn, and in situ hybridization studies have revealed the presence of 5-HT_3_ receptor mRNA in DRG neurons [305,309].

The 5-HT_3_ receptor is the only ionotropic serotonin receptor. It forms pentameric ion channels that are permeable to cations. Two subunits may contribute to the formation of ion channels: 5-HT_3A_ and 5-HT_3B_. 5-HT_3A_ homomeric and 5-HT_3A-3B_ heteromeric receptors are functional, but homomeric 5-HT_3B_ assemblies are nonfunctional [323]. Regardless of the unit composition, the activation of 5-HT_3_ receptors results in membrane depolarization [313,316,317].

Considering the antagonistic intracellular signaling mechanisms activated by the different 5-HT receptors, serotonin may have bidirectional functional effects on the terminals of various nociceptive primary afferent subpopulations. On the one hand, 5-HT_1_ receptors may attenuate; on the other hand, however, one may assume that 5-HT_2_, 5-HT_3_ and 5-HT_7_ receptors may facilitate neurotransmitter release from the axon terminals, exerting either anti- or pronociceptive effects on spinal nociceptive information processing. However, the slight membrane depolarization evoked by the 5-HT_2_, 5-HT_3_ and 5-HT_7_ receptors may actually attenuate transmitter release. Specifically, the application of serotonin to the hemisected spinal cord has been shown to evoke PAD in primary afferents, and this effect was mimicked by the application of 5-HT_3_ receptor agonists [180,324]. These authors also reported that the application of serotonin always depressed monosynaptic transmission from nociceptive primary afferents to spinal interneurons. If this is the case, it is difficult to answer the question of why so many 5-HT receptors are needed for the regulation of transmitter release from nociceptive primary afferents.

## 5. Conclusions

### 5.1. Synaptic Versus Volume Transmission

Neuroscientists like to describe the central nervous system as a complex network of neurons. According to this hypothesis, in morphologically and functionally defined parts of the central nervous system, there are microcircuits that are constructed from precisely wired excitatory and inhibitory neurons. The individual neurons forming the microcircuits are connected to each other via synapses; they release inhibitory and excitatory neurotransmitters into synaptic clefts, thus exciting and inhibiting each other via the activation of postsynaptic receptors, and as a result of this information processing, the microcircuit can perform certain neural functions. These microcircuits can communicate with each other locally and establish relationships with distant microcircuits via fiber projections. The communication pathways among different local and distant microcircuits tremendously increase the morphological and functional complexity of the central nervous system; however, if one can define the structure of the local microcircuits and their connections with others, one can ultimately develop a general idea of how nerve signals are processed and generate major functional properties of the central nervous system.

The transmission of nerve signals from nociceptive primary afferents to second-order sensory neurons in laminae I–II of the superficial spinal dorsal horn does not seem to follow this concept. In the case of the short-lasting low-frequency stimulation of nociceptive afferents evoking only a moderate amount of glutamate release from the axon terminals of nociceptive primary afferents, the activation of spinal neurons likely occurs via glutamatergic synaptic transmission. However, in the case of long-term peripheral noxious events evoking high-frequency discharges in nociceptive primary afferents such as peripheral inflammation, the release of pruritogen agents or neuropathy, excess glutamate that is released from nociceptive axon terminals may reach the perisynaptic zone of the postsynaptic membrane and activate the mGluR5-DAG-lipase complex, resulting in the release of 2-AG. In addition to glutamate’s various neuropeptides, BDNF and endomorphin can also be released from nociceptive axon terminals, which can dramatically change the activation patterns of local interneurons. The nonglutamate transmitters are released into the extracellular space where they diffuse and act on extracellular receptors via volume transmission, which cannot be described as the activation of precisely wired neural microcircuits. The extracellular release of these neurotransmitters can modulate the activities of various neurons in the vicinity (a few tens of a micrometer distance) of the site of transmitter release. All neurons within this area respond to transmitter release and carry proper extrasynaptic neurotransmitter receptors. Thus, the released neurotransmitters can modulate neural activities in a population of neurons defined by volume, and not by circuit connections. Furthermore, through the activation of neurotransmitter receptors such as cannabinoid, cytokine and chemokine receptors, astrocytes and microglia can also be activated, which can release biological substances such as 2-AG or BDNF that can act on neurons. In addition to neuron–neuron interactions, neuron–glia–neuron bidirectional communication pathways can also be activated, which may make the population responses of neurons even more complex. Thus, one may conclude from all these findings that spinal nociceptive information (pain and itch) processing should be regarded as population phenomena, as the activities of neuronal (and glial cell) populations in a volume of the superficial spinal dorsal horn, rather than only activities of precisely wired neuronal microcircuits.

### 5.2. Corelease of Neurotransmitters

There are two types of synaptic vesicles in the spinal terminals of nociceptive primary afferents, as follows: (1) Small clear vesicles that store glutamate. These vesicles are present in both peptidergic and nonpeptidergic axon terminals throughout laminae I–II. (2) Large dense-core vesicles that store the neuropeptides, BDNF and endomorphin. These vesicles can be observed only in peptidergic axon terminals in laminae I and IIo. High-resolution and sensitive immunohistochemical studies performed at the ultrastructural level have convincingly revealed that dense-core vesicles can store more than one neurotransmitter. Presumably most, if not all, of them contain CGRP, and many of them may store SP, GAL, SOM, NPP, NPY, BDNF and endomorphin in different combinations (Figure 2). Individual dense-core vesicles may contain only one neuropeptide (CGRP) or can store two, three or even four transmitters (Figure 2). Thus, in the case of strong long-lasting high-frequency noxious stimulation generated by various peripheral events resulting in peripheral sensitization, peptidergic nociceptive axon terminals always release more than one neurotransmitter. In addition to glutamate, they release CGRP in most cases (if not always), and depending on the nature of the peripheral noxious, pruritogenic or neuropathic events, they may also discharge other neurotransmitters. The coreleased neurotransmitters initiate both the synaptic (glutamate) and nonsynaptic (all other transmitters) transmission of nerve signals. Thus, the postsynaptic responses should be interpreted as a combination of postsynaptic events evoked by the activation of receptors sensitive to glutamate and the additional release of neuropeptides, BDNF or endomorphin. These activities can be even more complex than just the activation of a population of neurons in a volume of laminae I–II because, in addition to excitation, neuropeptides can evoke disinhibition, the inhibition of inhibitory interneurons by SOM via the activation of sst2A receptors, and the inhibition of excitatory interneurons by the activation of MOR, GALR1 or Y1 receptors by the released endomorphin, GAL or NPY, respectively. Thus, to understand the neural activities evoked by peripheral noxious or pruritogenic stimuli, proper knowledge about the expression and distribution of extrasynaptic receptors on the somatodendritic membrane of spinal interneurons and supraspinally projecting neurons can be as important as the corelease of neurotransmitters from peptidergic nociceptive axon terminals. Although nonpeptidergic axon terminals in lamina IIi release only glutamate, it is likely that the released neuropeptides and BDNF can diffuse from laminae I–IIo into lamina IIi; thus, they influence neuronal activities even in the termination area of nonpeptidergic afferents if the local interneurons in lamina IIi express the appropriate extrasynaptic receptors. Because of the relatively large distance between lamina IIo and lamina III, it is likely that the extrasynaptically released neurotransmitters in laminae I–IIo cannot diffuse into lamina III. Thus, the functional properties of local interneurons with dendrites and axons confined to lamina III or extending into deeper layers are likely not influenced by the extrasynaptic neurotransmitter cocktail in laminae I–II. However, dendrites and axons arising from neurons in laminae III–IV and extending into laminae I–II might be exposed to this mixture of neurotransmitters. Unfortunately, our present knowledge about these issues is quite limited.

### 5.3. Presynaptic Modulation of Synaptic Transmission

Both synaptic and extrasynaptic transmission between nociceptive primary afferents and secondary sensory neurons is under strong presynaptic modulation. Presynaptic axon terminals may express multiple neurotransmitter receptors (Table 1) that can be activated alone or in combination with others, and can attenuate or enhance neurotransmitter release depending on the transmitter substances discharged by the primary afferents, local interneurons or descending fibers.

The short-lasting low-frequency stimulation of nociceptive primary afferents presumably results in the release of glutamate only. The postsynaptic effect generated by the activation of ionotropic glutamate receptors anchored into the postsynaptic membrane can be controlled by the activation of multiple pre- and perisynaptic metabotropic glutamate receptors (Figure 1). First, mGluR4 and mGluR7 receptors, which are localized on the presynaptic membrane and can function as primary rate-limiting factors of glutamate release, can be activated. If the strength of stimulation increases, the release of glutamate can increase, and the released glutamate may reach the perisynaptic zone of the synaptic cleft, where excitatory amino acid transporters can take up excess glutamate back into the presynaptic axon terminal and/or into adjacent astrocytes. In addition, perisynaptic mGluR5 can also be activated, which, in collaboration with DAG-lipase-α, results in the release of 2-AG from postsynaptic dendrites. 2-AG can diffuse from the perisynaptic location into the extracellular space and, via volume transmission, activate extrasynaptic CB1 receptors expressed by neurons and glial cells in a certain volume of the dorsal horn around the glutamate-releasing synapses. Among other neural and glial elements, stimulated nociceptive axon terminals may also express CB1 receptors, the activation of which results in the closure of voltage-sensitive Ca^2+^ channels and the attenuation of glutamate release. Astrocytes that are adjacent to synapses and express excitatory amino acid transporters and/or CB1 receptors can thus take up glutamate, respond to 2-AG release, and release both glutamate and 2-AG into the extracellular space. Extracellular glutamate and 2-AG of astrocytic origin can bind to presynaptic mGluR2/3 and CB1 receptors, respectively. In this way, astrocytes may further attenuate glutamate release from presynaptic axons. Finally, owing to multiple negative feedback mechanisms, the activity of glutamatergic synapses made by nociceptive primary afferents can be attenuated or completely silenced.

In the case of peripheral inflammation, neuropathy or the release of pruritogen agents, specific groups of nociceptive afferents exhibit long-lasting and high-frequency firing, which substantially changes the neurochemical composition and functional properties of the spinal dorsal horn, including the presynaptic modulation of signal transmission from nociceptive primary afferents to spinal neurons. (i) Because CGRP-containing dense-core vesicles can be found in virtually all peptidergic nociceptive primary afferents, independent of the characteristics of the peripheral pathologic events, the excited peptidergic nociceptive afferents can release CGRP into the extracellular space. CGRP can excite spinal neurons and can also activate CLR–RAMP1, CTR–RAMP1 and other presynaptic CGRP receptors, which evoke positive feedback signals and may further increase the release of glutamate and neuropeptides, including CGRP, from the axon terminals. The release of additional glutamate and CGRP may further increase the excitation level of spinal neurons, which contributes to the development of a new functional state of the superficial spinal dorsal horn, called central sensitization. When SP is also released together with CGRP, in addition to the excitation of spinal neurons through NK and EP2 receptors, positive presynaptic feedback can be further increased by the activation of presynaptic EP receptors. Intracellular signaling events initiated by the activation of EP receptors further increase the release of neurotransmitters from nociceptive axon terminals, and further increase the intensity of central sensitization. In the case of pathologic peripheral events, when NGF can be a constituent of the “inflammatory soup”, together with glutamate, CGRP, and with or without SP, BDNF can also be released from peptidergic nociceptive primary afferents. (ii) Depending on the intensity of central sensitization, in addition to amino acid neurotransmitters, excited spinal neurons may also release other transmitters such as ATP. ATP discharged into the extracellular space activates P2X_2_ and P2X_2/3_ receptors expressed in axon terminals of non-peptidergic nociceptive primary afferents in lamina IIi. By increasing glutamate release from non-peptidergic primary afferents, ATP-presynaptic P2X receptor signaling further enhances the excitation level of spinal neurons, contributing to the development of central sensitization in lamina IIi. (iii) In the case of peripheral inflammation or tissue injury, nociceptive primary afferents also release cytokines and chemokines, which activate microglia via ligand–receptor interactions. Among several other changes, activated microglia substantially increases purinergic receptor expression, including the expression of P2X_4_ receptors. The activation of P2X_4_ receptors by ATP then initiates BDNF release from microglia. (iv) The BDNF released by axon terminals of peptidergic nociceptive primary afferents and activated microglia may act on both post- and presynaptic TrkB receptors, and thus may substantially increase the excitation level of neurons in the superficial spinal dorsal horn. This can sensitize neurons to glutamate via the phosphorylation of NMDA receptor subunits. It can also attenuate the hyperpolarizing effects of GABA_A_ and glycine receptors, or can even turn the original hyperpolarization into depolarization by downregulating the expression of KCC2, thus changing the intracellular concentration of Cl^−^ ions. In addition, BDNF can increase neurotransmitter release from both peptidergic and nonpeptidergic nociceptive axon terminals, which may also tremendously contribute to the development and maintenance of central sensitization. Seemingly the only substance that may evoke substantial effects, reducing the striking facilitatory effects of glutamate, CGRP, SP and BDNF, is endomorphin. Endomorphin is stored and presumably released, together with CGRP and SP, in the peptidergic nociceptive axon terminals. It activates both post- and presynaptic MORs, which attenuate neurotransmitter release from the axon terminals and inhibit postsynaptic neuron activation. The inhibitory effects of endomorphin can be enhanced by N/OFQ and endogenous opioid peptides released by spinal interneurons, which act on both pre- and postsynaptic NOP receptors, MORs and DORs. However, how the overwhelming facilitation evoked by glutamate, CGRP, SP and BDNF, resulting in central sensitization, can be inhibited by endomorphin, N/OFQ and endogenous opioid peptides is far from well-defined.

Axons of spinal GABAergic and glycinergic inhibitory neurons in laminae I–IV of the spinal dorsal horn may establish close appositions with the axon terminals of nociceptive primary afferents. The peptidergic axon terminals may establish contacts exclusively with GABAergic axons, whereas nonpeptidergic axon terminals may make close appositions with both GABAergic and glycinergic axon terminals. Because of the contrasting expression patterns of the NKCC1 cation–chloride cotransporter on peptidergic and nonpeptidergic terminals, the effects of the inhibitory amino acids on them can also differ. Because of the high expression and almost complete lack of NKCC1 expression on peptidergic and nonpeptidergic axon terminals, the intracellular Cl^−^ concentrations of peptidergic and nonpeptidergic axon terminals can be high and low, respectively. Thus, the activation of GABA_A_ receptors on peptidergic axon terminals can result in depolarization and the generation of PAD, and in some extreme cases, even in the generation of action potentials, increasing transmitter release, whereas GABAergic and glycinergic inputs may hyperpolarize the nonpeptidergic axon terminals and attenuate transmitter release from them. All these findings may lead to the surprising conclusion that the inhibitory amino acid GABA at the axon terminals of peptidergic nociceptive primary afferents can act in parallel with CGRP, SP and BDNF, increasing the possibility of transmitter release from peptidergic nociceptive axon terminals, and thus contributing to the development of central sensitization.

When monoaminergic nuclei in the hypothalamus, pons and medulla oblongata with neurons projecting into the superficial spinal dorsal horn are activated by the internal pain attenuation mechanisms of the central nervous system (e.g., in a fight or flight situation), axon terminals of the descending fibers may release dopamine, noradrenaline and serotonin within the superficial spinal dorsal horn. The peptidergic nociceptive axon terminals possess receptors for monoamines; α2A receptors for noradrenaline, D2-like receptors for dopamine, and 5-HT_1A/B_ receptors for serotonin. The activation of these receptors results in the attenuation of transmitter release from the axon terminals, representing a powerful compensation for the facilitatory effects of the CLR–RAMP1, CTR-RAMP1, TrkB and EP receptors, activated by CGRP, BDNF and PGE2, respectively. However, the effect of serotonin on transmitter release from nociceptive axon terminals is not clear. That is, the axon terminals of nociceptive primary afferents may also express other serotonin receptors, such as 5-HT_2A_, 5-HT_3_ and 5-HT_7_, the activation of which may increase transmitter release from the axon terminals, thus generating pronociceptive effects. This possible bidirectional effect of serotonin causes challenges in the interpretation of how serotonin may influence neurotransmitter release from nociceptive axon terminals and spinal nociceptive information processing in general. A thorough investigation of the expression patterns of serotonin receptors in different functional states of the superficial spinal dorsal horn is needed to elucidate this mechanism.

## Figures and Tables

**Table 1:** Receptors playing roles in presynaptic modulation of transmitter release from axon terminals of nociceptive primary afferents in laminae I–II of the superficial spinal dorsal horn.

Receptor	Ligand	Localization of the Receptor	Coupled Intracellular Protein	Effect of the Activation of the Receptor
mGluR4 mGluR7	Glutamate	Presynaptic membrane	Gi/o	Attenuation of transmitter release
mGluR2 mGluR3	Glutamate	Extrasynaptic	Gi/o	Attenuation of transmitter release
mGluR5	Glutamate	Perisynaptic	Gq/11 DAG-lipase	Activation of retrograde endocannabinoid signaling
CB1	2–AG	Extrasynaptic on both peptidergic and nonpeptidergic terminals	Gi/o	Attenuation of transmitter release
CLR–RAMP1 CTR-RAMP1	CGRP	Extrasynaptic on peptidergic terminals	Gs	Enhancement of transmitter release
EP1, EP3, EP4 (?)	PGE2	Extrasynaptic	G_q/11_, G_s_, G_i_ (?)	Enhancement of transmitter release
GALR2	GAL	Extrasynaptic on peptidergic terminals	Gi/o, Gq/11	Enhancement of transmitter release (?)
Y2	NPY	Extrasynaptic on both peptidergic and nonpeptidergic terminals	Gi/o	Attenuation of transmitter release
TrkB	BDNF	Extrasynaptic on both peptidergic and nonpeptidergic terminals	Tyrosine residues	Enhancement of transmitter release
GABA_A_	GABA	Extrasynaptic on both peptidergic and nonpeptidergic terminals	Ionotropic	May generate hyperpolarization or PAD depending on the expression of NKCC1
GABA_B_	GABA	Extrasynaptic on both peptidergic and nonpeptidergic terminals?	Gi/o	Attenuation of transmitter release
GlyR	Glycine	Extrasynaptic on nonpeptidergic terminals	Ionotropic	Attenuation of transmitter release
MOR	Endomorphin, enkephalin, β-endorphin	Extrasynaptic on peptidergic terminals	Gi/o	Attenuation of transmitter release
DOR	Enkephalin, β-endorphin	Extrasynaptic on nonpeptidergic terminals	Gi/o	Attenuation of transmitter release
KOR	Dynorphin	Extrasynaptic	Gi/o	Attenuation of transmitter release
NOP	N/OFQ peptide	Extrasynaptic on both peptidergic and nonpeptidergic terminals	Gi/o	Attenuation of transmitter release
P2X_3_, P2X_2/3_	ATP	Extrasynaptic on nonpeptidergic terminals	Ionotropic	Enhancement of transmitter release
α2A	Noradrenaline	Extrasynaptic on peptidergic terminals	Gi/o	Attenuation of transmitter release
D2, D3, D4	Dopamine	Extrasynaptic	Gi/o	Attenuation of transmitter release
5-HT_1A_ 5-HT_1B_	Serotonin	Extrasynaptic on peptidergic terminals	Gi/o	Attenuation of transmitter release
5–HT_2A_	Serotonin	Extrasynaptic on both peptidergic and nonpeptidergic terminals	Gq/11	Enhancement of transmitter release (?)
5–HT_3_	Serotonin	Extrasynaptic on both peptidergic and nonpeptidergic terminals	Ionotropic	Enhancement of transmitter release (?)
5–HT_7_	Serotonin	Extrasynaptic	Gs	Enhancement of transmitter release (?)
